# Peptide nucleic acid-zirconium coordination nanoparticles

**DOI:** 10.1038/s41598-023-40916-w

**Published:** 2023-08-30

**Authors:** Özgür Öztürk, Anna-Lina Lessl, Miriam Höhn, Stefan Wuttke, Peter E. Nielsen, Ernst Wagner, Ulrich Lächelt

**Affiliations:** 1 Department of Pharmacy and Center for NanoScience (CeNS), LMU Munich, 81377 Munich, Germany; 2 Department of Genetic and Bio Engineering, Alanya Alaaddin Keykubat University, Antalya, Türkiye; 3 Basque Center for Materials (BCMaterials), Leioa, Spain; 4 Ikerbasque, Basque Foundation for Science, Bilbao, Spain; 5 Department of Cellular and Molecular Medicine, University of Copenhagen, Copenhagen, Denmark; 6 Department of Pharmaceutical Sciences, University of Vienna, Vienna, Austria

**Keywords:** DNA and RNA, Drug delivery, Nanoparticles, Organic-inorganic nanostructures

## Abstract

Ideal drug carriers feature a high loading capacity to minimize the exposure of patients with excessive, inactive carrier materials. The highest imaginable loading capacity could be achieved by nanocarriers, which are assembled from the therapeutic cargo molecules themselves. Here, we describe peptide nucleic acid (PNA)-based zirconium (Zr) coordination nanoparticles which exhibit very high PNA loading of $>\,94\%$ w/w. This metal-organic hybrid nanomaterial class extends the enormous compound space of coordination polymers towards bioactive oligonucleotide linkers. The architecture of single- or double-stranded PNAs was systematically varied to identify design criteria for the coordination driven self-assembly with Zr(IV) nodes at room temperature. Aromatic carboxylic acid functions, serving as Lewis bases, and a two-step synthesis process with preformation of $Zr_{6} O_{4} (OH)_{4}$ turned out to be decisive for successful nanoparticle assembly. Confocal laser scanning microscopy confirmed that the PNA-Zr nanoparticles are readily internalized by cells. PNA-Zr nanoparticles, coated with a cationic lipopeptide, successfully delivered an antisense PNA sequence for splicing correction of the $\beta$-globin intron mutation IVS2-705 into a functional reporter cell line and mediated splice-switching via interaction with the endogenous mRNA splicing machinery. The presented PNA-Zr nanoparticles represent a bioactive platform with high design flexibility and extraordinary PNA loading capacity, where the nucleic acid constitutes an integral part of the material, instead of being loaded into passive delivery systems.

## Introduction

Metal-organic frameworks (MOFs) are inorganic-organic hybrid materials composed of metal ions or metal clusters and organic linkers with Lewis base functions. Coordination driven self-assembly of the organic linkers and metal nodes leads to the creation of highly ordered, in many cases porous, two or three dimensional frameworks^1,2^. Although MOFs are overwhelmingly crystalline structures, non-crystalline MOFs such as amorphous MOFs, MOF liquids, MOF glasses and other coordination polymers are reported in the literature^3^. The versatile assembly strategy enables the creation of hybrid materials with a variety of features by selecting suitable building units^4^. This design flexibility creates an enormous compound space with a high number of generated MOFs and coordination polymers for diverse purposes^5–8^. In the context of biomedical applications, MOFs and coordination polymers have been designed as carriers for small molecular drugs or biomolecules as well as frameworks with photo-sensitizing, radiation-enhancing and bio-imaging properties^9–17^. UiO-66, constructed from hexanuclear zirconium oxide clusters $(Zr_6 O_4 (OH)_4)$ and terephthalic acid (TPA), belongs to the set of most intensively researched MOFs^18,19^. Due to the straight-forward synthesis, exceptional stability and very high surface area, UiO-66 and derivatives were also frequently assessed for utilization as drug carriers^20^. One generally critical parameter for the application of nanopharmaceuticals is represented by the potential toxicity of the material. In case of MOFs, the tolerability of the individual components as well as the nanotoxicity of assembled particles have to be taken into consideration^21^. Zr is present in biological systems and has moderately low toxicity, as found in histological and cytological studies^22^. Zr based MOFs generally possess low toxicity related to the metal component, although degrees of biocompatibility can vary depending on the organic linker components and individual structures^23,24^. A strategy to overcome some toxicity issues is the utilization of well tolerated endogenous organic ligands, such as amino acids^25–27^, peptides^28–30^, proteins^31,32^ or nucleobases^33,34^ for the generation of “Bio-MOFs”^35,36^. However, even in case of well-tolerated nanomaterials, a high drug loading capacity and minimal exposure of patients to excessive carrier material is favorable to prevent adverse reactions. Theoretically, maximal drug loading could be achieved by nanomaterials which are formed by the bioactive entities themselves. Metal-organic nanopharmaceuticals built from drug molecules with Lewis base functions which assemble into coordination nanoparticles with metal ions approximate the envisioned nanocarriers with maximal loading capacity^37,38^. While this concept has already been realized with low molecular weight therapeutics^39–42^, coordination polymers containing linkers based on bioactive biomolecules or synthetic analogs have not been reported.

Previously reported strategies for the generation of nucleic acid containing MOFs were mostly based on binding to internal or external MOF surfaces, and biomineralization during MOF assembly. Both covalent and noncovalent interactions have been used to functionalize MOFs with oligonucleotides^43^. For example, the zirconium MOF UiO-66-N3 which contains organic linkers with azido group, was covalently functionalized with dibenzocyclooctyne (DBCO) modified DNA oligonucleotides via strain-promoted click reaction^10^. Furthermore, UiO-68 containing amino-triphenyldicarboxylic acid as ligand was used for non-covalent binding of siRNA to the surface^44^. Similarly, the zirconium MOFs, NU-1000 and PCN-222/MOF-545, were functionalized with phosphate terminated oligonucleotides via coordinative interactions^45^. Independent of the individual interaction mode, critical parameters for the incorporation of oligonucleotides into internal cavities are the pore dimensions which determine the accessibility to the inner MOF matrix^46,47^. Alternatively, large biomolecules including nucleic acids, have been encapsulated into MOFs by biomineralization during framework assembly^48,49^. In all these cases, oligonucleotides represent guest molecules in distinct MOF materials. In contrast, using nucleic acids as essential framework components has not been realized so far, although it is suggested to be favorable to increase the loading capacity and simplify manufacturing. Peptide Nucleic Acids (PNAs) are synthetic nucleic acid analogs which contain a peptide backbone of N-(2-aminoethyl)-glycine units with nucleobases attached via carbonyl methylene linkers^50^. Similar to natural nucleic acids, PNAs bind with high affinity and specificity to complementary DNA, RNA or PNA sequences. In fact, the affinities and binding strengths of complementary PNA-PNA, PNA-DNA or PNA-RNA duplexes are higher and less affected by ionic stress then duplexes from analog DNA or RNA sequences^51,52^. PNAs represent a powerful synthetic nucleic acid technology due to (1) their stable and strong binding ability, (2) good mismatch sequence discrimination, (3) synthetic accessibility and flexibility, and (4) resistance to degradation by nucleases and proteases^53–55^. Besides bioanalytical applications such as nucleic acid probing, in situ hybridization and PCR modulation^56–59^. PNAs can function as antisense oligonucleotides to silence gene expression or modulate cellular mRNA splicing^60,61^. Similar to other nucleic chemistries, cellular delivery represents a major challenge for antisense applications of PNAs. Despite the neutral charge and lipophilic character, PNAs do not cross the cellular membrane efficiently. For this reason, PNAs were conjugated to cell-penetrating peptides, formulated in delivery systems, or chemically modified to enable cellular entry^62–65^. Here we present new kinds of metal-organic coordination nanoparticles based on PNA linkers and zirconium(IV) (PNA-Zr nanoparticles). To the best of our knowledge, the presented material is the first example of coordination polymers built from nucleic acid analogs as organic linker component. For the application as nanocarrier, PNA-Zr nanoparticles feature a very high loading capacity, are readily internalized by cells and can transport functional PNAs for splicing correction via sequence specific interference with endogenous mRNA splicing processes.

## Results

### Development of PNA-Zr particles

The design and selection of suitable organic linkers is a key step in MOF synthesis and critically impacts the MOF properties and characteristics after assembly. The linkers require Lewis base functions containing N- or O-donor atoms such as carboxylic acids or aromatic N-heterocycles. Most established MOF linkers possess a rigid structure with restricted flexibility due to unsaturated bonds, cycles or arenes connecting the separated Lewis base functions. In order to establish a platform for the creation of PNA-Zr coordination nanoparticles, different PNA linker architectures were designed to mimic characteristics of established MOF linkers: (1) aromatic carboxylic acids were attached at the PNA termini to provide Lewis base functions at distant positions; (2) single- and double-stranded PNAs were used to modulate the linker rigidity. Unfortunately, most common MOF syntheses under solvothermal conditions are not compatible with double-stranded PNAs, since PNA duplexes melt and dissociate at the required high temperatures. Therefore, a strategy for MOF synthesis at room temperature (RT) had to be chosen.

**Scheme 1:**
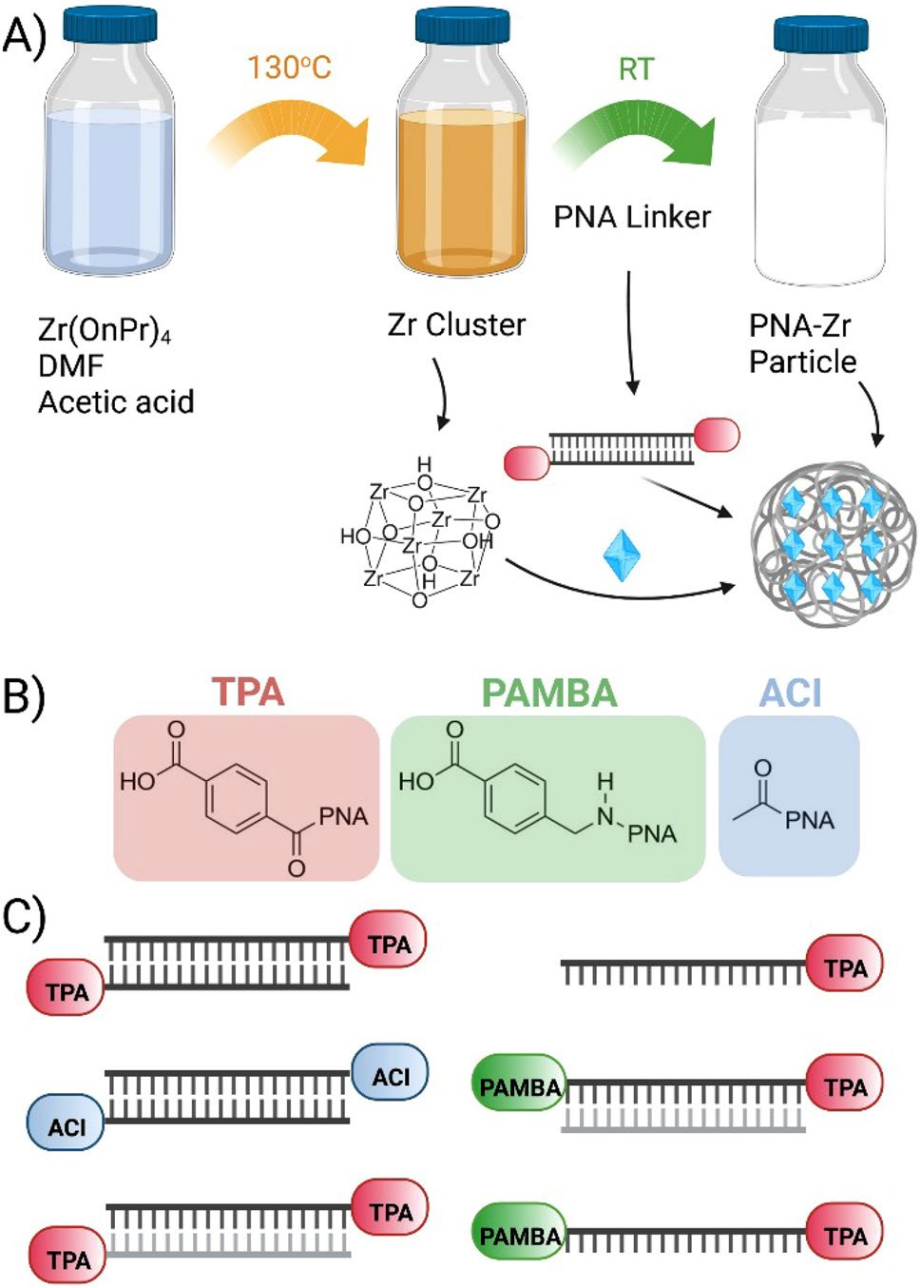
Synthesis process of particles, PNA modifications and linker architectures. (**A**) Synthesis of PNA-Zr particles via a two-step process. (**B**) PNA end-modifications: left terephthalic acid (TPA, red), center para-aminomethylbenzoic acid (PAMBA, green) and right acetic acid (ACI, blue). (**C**) PNA linker architectures. Created with BioRender.com.

The lab of Omar Farha developed a RT synthesis of UiO-66, which is constructed from hexanuclear zirconium oxide clusters $Zr_6 O_4 (OH)_4$ and TPA^66^. In this strategy, classical one-pot solvothermal MOF synthesis is dissected into the sequential steps of (1) preforming secondary building units from zirconium propoxide $Zr(OnPr)_4$ at high temperatures in presence of excessive acetic acid, followed by (2) addition of the TPA linker and incubation at RT. This work served as basis for the adaption towards Zr-coordination nanoparticles with temperature sensitive PNA linkers. The strategy for the formation of the particles is illustrated in Scheme 1A. Analog to the work by DeStefano et al.^66^, coordination particles are formed in a two-step process^67^. First, Zr nodes are generated by incubating a solution containing $Zr(OnPr)_4$, DMF and acetic acid at $130\,^{\circ }\hbox {C}$ for 2 h. Then the solution of metal nodes is cooled to RT and added to a PNA linker solution at a 1:1 molar ratio of linker to Zr. In case of double-stranded PNA linkers, an initial annealing step (heating to $90\,^{\circ }\hbox {C}$, cooling to RT) was carried out to ensure duplex formation before nanoparticle synthesis. The assembly mixture was incubated for 24 h at RT and the formed PNA-Zr particles were isolated as a white precipitate by centrifugation. As illustrated by Scheme 1C, several PNA linkers with different modifications were designed; para-aminomethylbenzoic acid (PAMBA) and TPA end modifications were introduced at the C- (PAMBA) and N- (TPA) termini of PNA sequences to serve as aromatic carboxylic acid functions for coordinative interactions with metal nodes. In contrast, acetylated (ACI) N-termini served as controls without Lewis base function. Table 1 summarizes the PNA sequences that were evaluated and the capability to serve as linkers for particle formation with Zr nodes.

**Table 1:** Summary of investigated PNA linkers. The linkers are composed of either double-stranded (ds) or single strand (ss) PNA architectures. Successful particle formation is indicated by ‘YES’ (observed) or ‘NO’ (not observed).

PNA sequences	Description	Architecture	Particle formation
GC-ACI	Palindromic	ds	*NO*
GC-TPA	Palindromic	ds	*NO*
CATG-ACI	Palindromic	ds	*NO*
CATG-TPA	Palindromic	ds	*NO*
GCATGC-ACI	Palindromic	ds	*NO*
GCATGC-TPA	Palindromic	ds	**YES**
CAGTACTG-ACI	Palindromic	ds	*NO*
CAGTACTG-TPA	Palindromic	ds	**YES**
CGTGAC-TPA + GTCACG-TPA	Non-palindromic	ds	**YES**
CGTGAC-TPA + GTCACG	Non-palindromic	ds	minor formation
PAMBA-CGTGAC-TPA + GTCACG	Non-palindromic	ds	**YES**
PAMBA-CGTGAC-TPA	Non-palindromic	ss	**YES**
PAMBA-GCAGCT-TPA	Non-palindromic	ss	**YES**
PAMBA-CCTCTTACCTCAGTTACA-TPA	Non-palindromic (705 SSO)	ss	**YES**

It is important to note that PNA linkers generally were purified by size exclusion chromatography without acid additives in the solvent, since it was observed that HCl salts of PNAs negatively affect particle formation. Palindromic PNA sequences with modification of the N-terminus (TPA or ACI), which assemble into duplexes due to self-complementarity, served as simple models of double-stranded linkers. The shortest palindromic sequence that formed particles with Zr nodes exhibited a length of 6 PNA monomer units, but only if the N-terminus was modified with TPA. In contrast, palindromic 4- and 2-mers did not form particles regardless of TPA or ACI end-modifications. It is suggested that the low melting temperature of the shorter PNA sequences is responsible for a lack of dimerization and formation of linkers with two coordinative end functionalities^68^.

The observation that acetylation generally prevented particle formation with Zr nodes indicates the need of TPA as Lewis base end modification. Consistently, the combination of two complementary, non-palindromic 6-mer PNAs with TPA at the N-termini was also able to form particles, but an exact 1:1 molar ratio of both PNAs was essential. PNA sequences equipped with two metal coordination sites at the N- (TPA) and C- (PAMBA) termini were suitable for particle formation, independent whether it was used as duplex with an additional complementary strand (without PAMBA and TPA) or as single-stranded linker alone. The combination of a PNA sequence with one TPA at the N-terminus and the complementary PNA without modification, which leads to a double-stranded PNA with only one aromatic carboxylic acid end function, caused a minor formation of solid material. The observations show that both single- and double-stranded PNAs are able to form coordination particles with Zr nodes, but two carboxylic acid end functions are required to yield substantial amounts.

### Characterization of PNA-Zr particles

**Figure 1:**
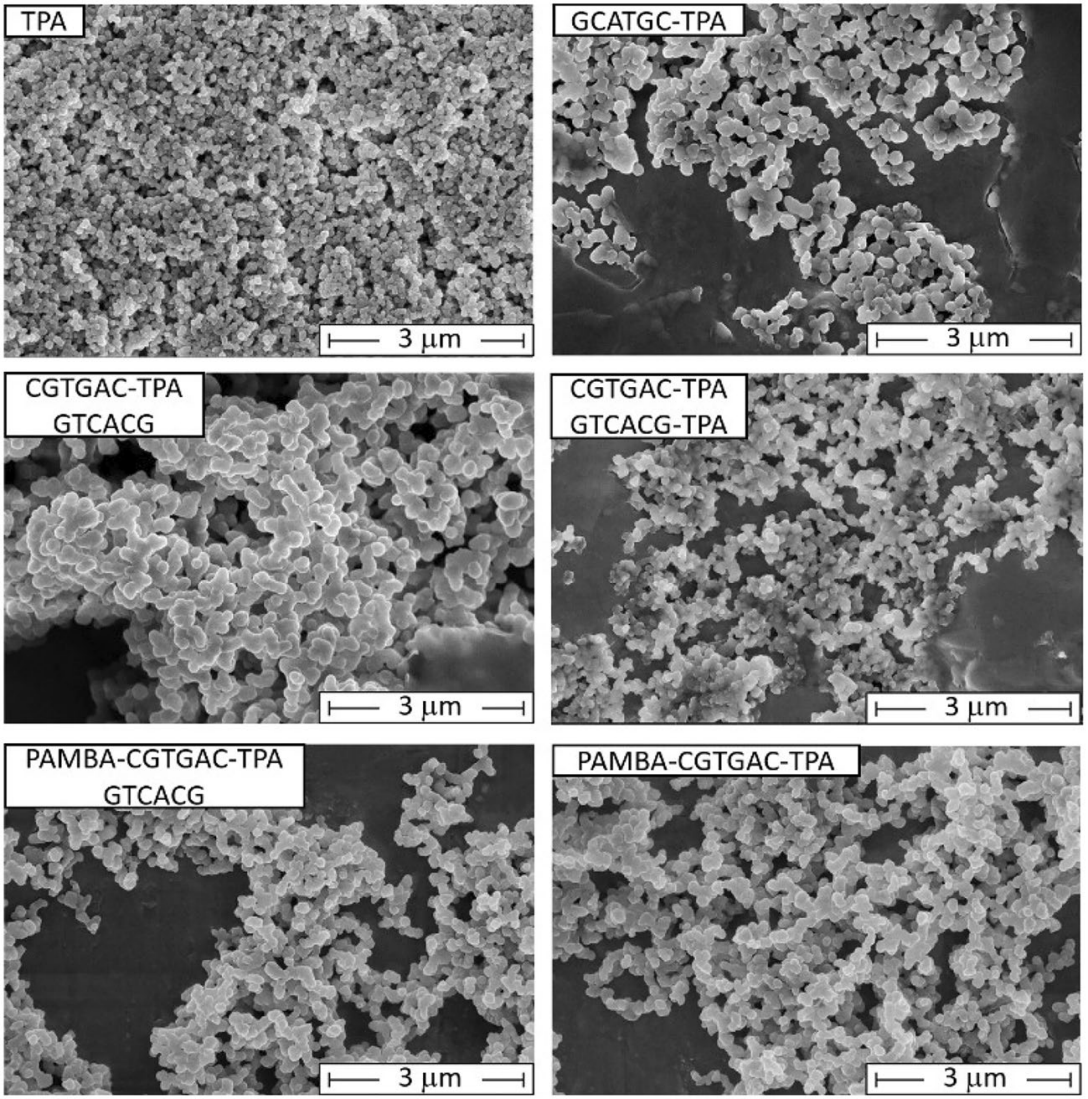
Representative SEM images of PNA-Zr nanoparticles. The sequences of individual PNA linkers is specified in the upper left corner of the images. TPA leading to the formation of UiO-66 was used as a reference (scale bars = 3 $\upmu$m).

The obtained PNA-Zr particles were characterized by a set of physicochemical characterization techniques. Particle size, morphology and surface charge were determined by scanning electron microscopy (SEM), dynamic light scattering (DLS) and electrophoretic light scattering (ELS). Representative SEM images (Fig. 1) show small (between 150-250 nm diameter) mostly spherical particles formed with different PNA linkers. DLS measurements (Fig. S1 in the Electronic Supplementary Material, ESM) showed larger particles with z-averages between 500 to 1000 nm. The deviation can be explained by a high sensitivity of DLS towards aggregates and larger particles as well as the fact that DLS detects hydrodynamic particle sizes in solution, whereas SEM measurements are carried out with dried samples^69^. Electrophoretic light scattering measurements determined negative zeta potentials between $-5$ to $-10$ mV.

**Figure 2:**
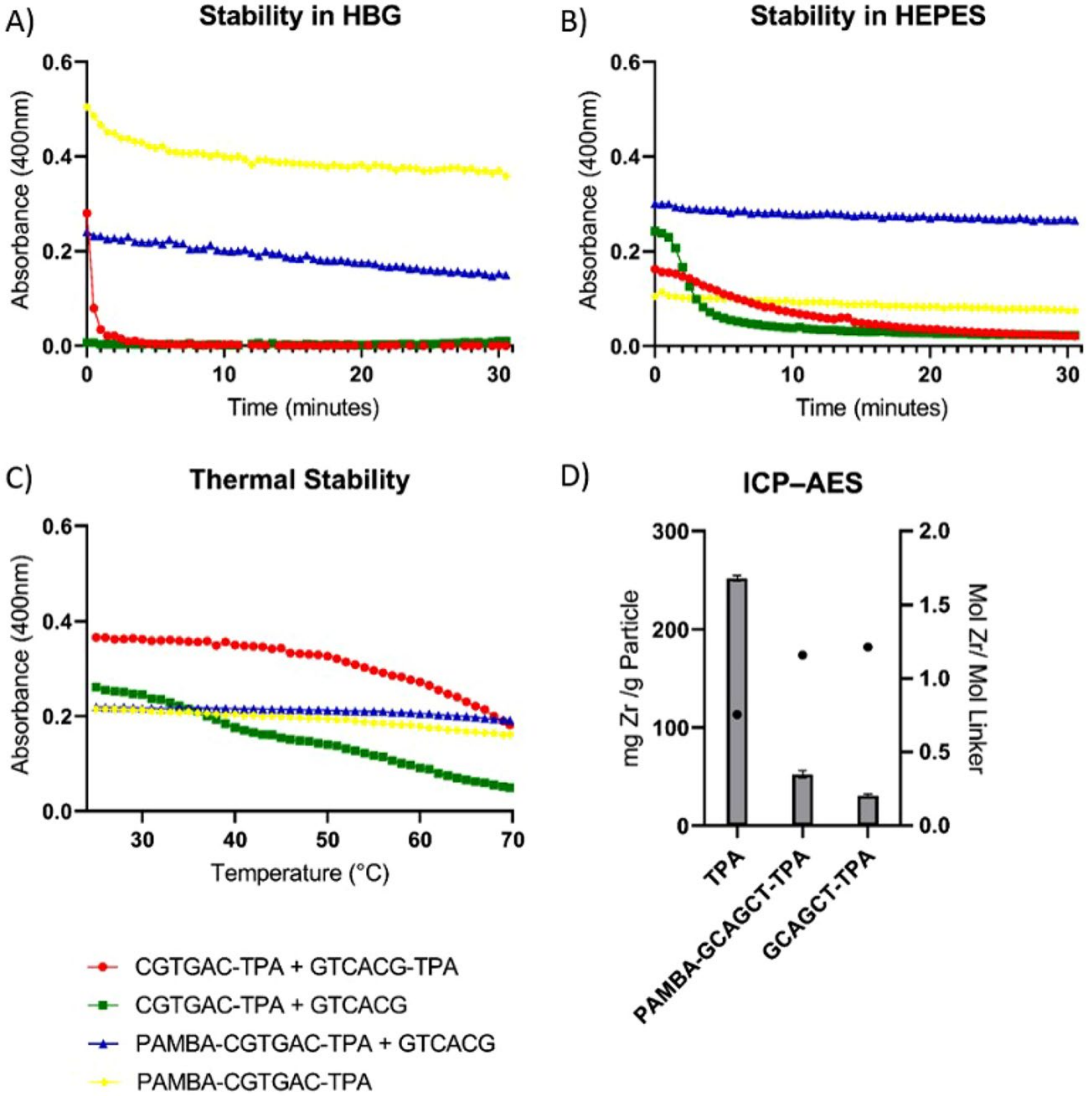
Stability of PNA-Zr particles and ICP-AES. Stability of PNA-Zr particles determined by measurement of optical density (400 nm) after incubation in (**A**) HBG, (**B**) HEPES or (**C**) at increased temperatures. PNA linker architectures are indicated by colors (see legend below) (**D**) Zr content of PNA-Zr particles determined by ICP-AES and resulting molar ratios calculated under consideration of the molecular weight of the linkers.

Thermal stability of PNA-Zr particles were investigated by monitoring the optical density (400 nm) of nanoparticle suspensions exposed to a temperature gradient. In general, turbidity or light scattering measurements are carried out at a wavelength in the visible spectrum, which does not overlap with absorption maxima of individual components; PNAs, similar to other nucleic acid analogs, have an absorption maximum in the UV spectrum at 260 nm^70,71^. As seen in Fig. 2C, PNA-Zr particles are stable at RT regardless of the PNA linker architecture. At higher temperatures, particles built from double-stranded linkers with TPA modification on both strands disassemble at elevated temperatures, which can be recognized as drop of the optical density. A reasonable explanation is that the PNA duplexes melt at the increased temperatures, which results in disassembly of the linkers with TPA function on both ends. In contrast, particles containing linkers with PAMBA and TPA in a single strand did not show similar temperature sensitivity. These results suggest two key characteristics of the PNA-Zr nanoparticles: (1) two aromatic carboxylic acid functions, located at both ends of the PNA linkers, are essential; and (2) double-stranded linkers with aromatic carboxylic acid functions separated on both strands are sensitive towards linker disassembly via duplex melting. The stability of PNA-Zr particles in biological buffers, isohydric HEPES (20 mM, pH 7.4) as well as isohydric and isotonic HBG (20mM HEPES, pH 7.4, 5% glucose), was investigated with the same approach (Fig. 2A,B). PNA-Zr particles were added to HBG or HEPES and the optical density was monitored at 400 nm over time. Similar to the results of the thermal stability study, particles containing linkers with two aromatic carboxylic acid functions in a single PNA strand exhibited higher stability, whereas particles composed of double-stranded linkers with TPA modifications in separate strands dissociated.

X-ray diffraction (XRD) was used to elucidate the presence of crystallinity in representative PNA-Zr nanoparticles built from double-stranded (palindromic GCATGA-TPA) or single-stranded (non-palindromic PAMBA-CGTGAC-TPA and PAMBA-CCTCTTACCTCAGTTACA-TPA) linkers (Fig. S2 in the ESM). In contrast to the characteristic reflections obtained with UiO-66, no indication for crystallinity of PNA-Zr nanoparticles was found by XRD; therefore, rather amorphous structures of PNA-Zr coordination particles are suggested. Further characterization by nitrogen sorption determined a surface area of 300 m$^{2}$/g for PNA-Zr nanoparticles built from GCATGA-TPA and 36 m$^{2}$/g for nanoparticles from PAMBA-CGTGAC-TPA (Fig. S3, Table S3 in the ESM)^72^. The absorption isotherm of PAMBA-CGTGAC-TPA showed similarity to a type II absorption isotherm indicating a non-porous or macro-porous structure, whereas GCATGA-TPA could not be assigned to any characteristic type^73,74^.

Next, the Zr content of PNA-Zr particles was determined by inductively coupled plasma atom emission spectrometry (ICP-AES, Fig. 2D. A content of 52 mg/g Zr was determined for PAMBA-CGTGAC-TPA particles and 31 mg/g Zr for GCATGA-TPA particles, which reflects a very high PNA content of 94.8% or 96.9%, respectively. Based on the obtained results and the molecular weights of PNA linkers, the stoichiometry of the components in the particle assembly was calculated. Molar ratios of Zr to linker of around 1.2:1 were determined for both representative types of PNA-Zr nanoparticles, which is close to the 1:1 ratio reported for UiO-66^18^.

### Cellular uptake of PNA-Zr particles

**Figure 3:**
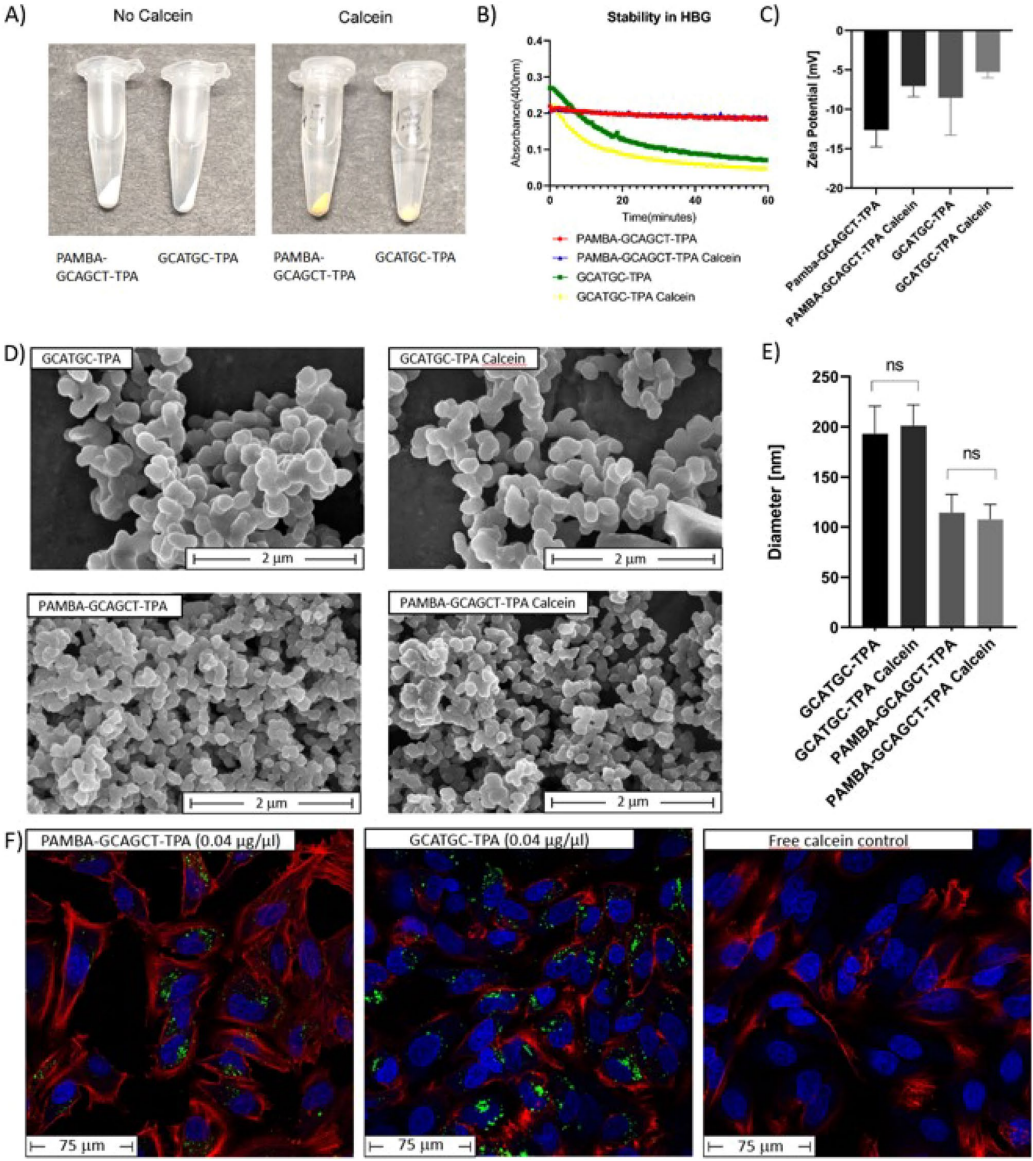
Calcein loaded particles (**A**) Representative images of PNA-Zr particles before and after calcein loading. (**B**) Stability of calcein loaded and unlabeled PNA-Zr particles in HBG as determined by measurement of optical density (400 nm). (**C**) Zeta potential of calcein loaded particles measured by ELS. (**D**) Representative SEM images of calcein loaded and unlabeled PNA-Zr nanoparticles. The individual linkers used for particle assembly are stated in the upper left corner of each image (scale bars = 2 $\upmu$m). (**E**) Evaluation of particle sizes obtained by SEM imaging (diameter, nm). n = 30 random particles were evaluated for the determination. (**F**) CLSM images of HeLa pLuc/705 cells treated with calcein (green fluorescence) loaded PNA-Zr particles. Cells were incubated for 4 h with calcein loaded particles (0.04 $\upmu$g/$\upmu$L) or free calcein at the concentration corresponding to the maximal calcein content of particles (0.0005 $\upmu$g/$\upmu$L). Subsequently, the cells were incubated for additional 4 h with fresh medium before imaging. Cell nuclei were stained with DAPI (blue), and actin was visualized by staining with phalloidine-rhodamine (red).

The fluorescent dye calcein, which contains Lewis base functions and can form chelates with metal ions, was utilized for labeling of PNA-Zr particles by coordinative interaction^42,75^. For this, PNA-Zr particles were incubated with an aqueous calcein solution (0.25 mM) at RT. At the end of the labeling procedure, a clear color change from white to orange particles was observed (Fig. 3A). Direct comparison of labeled and unlabeled PNA-Zr particles in the established stability assay confirmed that calcein loading did not affect the stability characteristic in HBG (Fig. 3B). Calcein-loaded PNA-Zr particles based on GCATGA-TPA, degraded within 1 h in HBG, while PAMBA-CGTGAC-TPA particles did not show significant particle disassembly. Further, the nanoscopic appearance of labeled and unlabeled PNA-Zr particles was compared by SEM imaging. As seen in Fig. 3D,E, calcein loading did not significantly impact the size and shape of the particles. ELS measurements (Fig. 3C) indicated a slight increase of the zeta potential upon calcein loading, but the overall charge of the nanoparticles remained negative. The results showing unaffected particle properties were essential requirements for the following determination of cellular uptake via confocal laser scanning microscopy (CLSM) imaging of calcein-loaded PNA-Zr nanoparticles (Fig. 3F).

HeLa pLuc/705 cells were incubated for 4 h with labeled particles based on double-stranded palindromic GCATGA-TPA or single-stranded PAMBA-CGTGAC-TPA linkers, followed by a medium change and additional 4 h incubation. CLSM imaging showed that both types of PNA-Zr nanoparticles were internalized by the cells, whereas free calcein at the concentration corresponding to the maximal particle loading, could not be detected intracellularly. This indicates that PNA-Zr nanoparticles are suitable for the intracellular transport of impermeable compounds and that the particles did not dissociate in the cell culture medium.

### Antisense activity of PNA-Zr nanoparticles

Since PNAs can be utilized as antisense oligonucleotides which modulate the cellular mRNA splicing process, the capability of PNA-Zr coordination nanoparticles to transport functional splice-switching PNAs into cells was assessed. Kang et al. generated HeLa cells with stable expression of a luciferase reporter (pLuc/705) for detecting corrections of aberrant $\beta$-globin splicing which is caused by an intron mutation (IVS2-705)^76,77^. Here, we generated PNA-Zr particles containing the PNA sequence against the location of IVS2-705, which is able to induce splicing correction, if delivered into the nucleus of cells. With this test system, successful cellular delivery and splice-switching activity can be detected via bioluminescence due to the induction of functional luciferase expression^77,78^. A single-stranded PNA linker with the sequence PAMBA-CCTCTTACCTCAGTTACA-TPA (705 SSO) has been used for particle formation via the established two step formation process.

**Figure 4:**
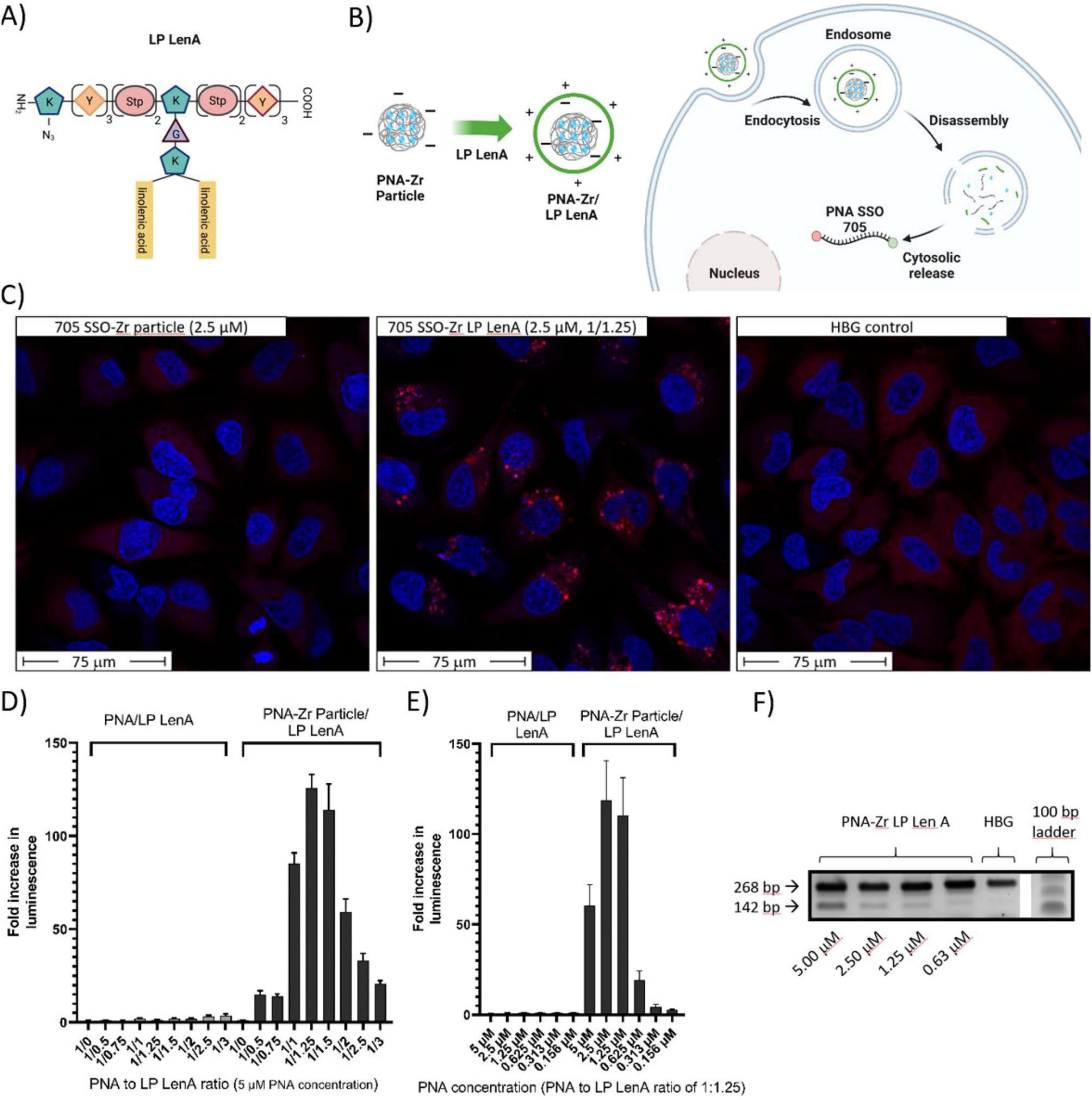
Delivery of functional PNA by PNA-Zr coordination nanoparticles (**A**) Structure of LP LenA composed of: N-terminal azidolysine, tyrosine (Y), lysine (K), succinyl-tetraethylenepentamine (Stp), glycine (G) and linolenic acid. (**B**) Schematic illustration of LP-LenA coatied PNA-Zr nanoparticles and the intracellular delivery process. Created with BioRender.com. (**C**) CLSM images of HeLa mRuby3/gal8 cells treated with 705 SSO-Zr particles (left), LP LenA coated 705 SSO-Zr particles (1/1.25 ratio, middle) and HBG control (right). mRuby3/gal8 cells were incubated for 8 h with particles (2.5 $\upmu$M PNA content). Subsequently, the cells were incubated for additional 4 h with fresh medium before imaging. (**D**) Splice-switching activity of 705 SSO-Zr nanoparticles (5 $\upmu$M PNA) at different PNA/LP LenA coating ratios in HeLA pLuc/705 cells. (**E**) Splice-switching activity of 705 SSO-Zr nanoparticles (1/1.25 PNA/LP LenA ratio) at different PNA concentrations in HeLA pLuc/705 cells. (**F**) RT-PCR of $\beta$-globin IVS2 from HeLa pLuc/705 cells treated with PNA-Zr/LP LenA at ratio 1/1.25. Total RNA was isolated 48 h after treatments and $\beta$-globin IVS2 was amplified. Band at 268 bp represents the aberrant splicing product; band at 142 bp corresponds to the product after splice-switch. The image has been cropped for clear presentation, the original gel is provided in Fig. S12 in the ESM.

FT-IR spectroscopy confirmed the presence of the relatively long 705 SSO PNA in the derived PNA-Zr particles (Fig. S4 in the ESM). By quantifying the amount of encapsulated PNA after decomposition of PNA-Zr particles, an encapsulation efficiency of 85.2%, in relation to the PNA amount used for synthesis, was determined. HeLa pLuc/705 cells were treated with the PNA-Zr nanoparticles and the occurrence of splice-switching was assessed by determination of luciferase activity via bioluminescence measurements after 48 h. In preliminary experiments, the bare PNA-Zr particles were not able to mediate detectable splicing correction. Since cellular uptake of PNA-Zr particles was demonstrated previously, insufficient endosomal escape, which generally represents a critical hurdle for the delivery of biomacromolecules, was suspected to be responsible. To improve the endosomal escape and enable transport to the intracellular target sight, PNA-Zr particles were coated with a cationic artificial peptide (Fig. 4A,B). The lipopeptide (LP) LenA (K_ε_(N_3_)-Y_3_-Stp_2_-K_ε_[G-K_α,ε_(linolenic acid)_2_]-Stp_2_-Y_3_) was previously shown to be a suitable agent for the delivery of splice-switching phosphorodiamidate morpholino oligomers (PMOs) into HeLa pLuc/705 cells after covalent conjugation^78^.

Since PNA-Zr particles possess a negative zeta potential as well as coordinatively unsaturated metal sites at the external surface, coating with the oligo(ethanamino)amide LP LenA is suggested a feasible strategy for post-synthetic nanoparticle modification. SEM imaging determined the morphology of the particles which were assembled from the long 705 SSO PNA linkers (Fig. S5 in the ESM). The results demonstrated that 705 SSO-Zr nanoparticles were mostly spherical and exhibited small sizes between 150-250 nm in diameter. After coating with LP LenA, the spherical shape was preserved, though a slight increase in size seemed to be observable. The successful modification of the 705 SSO-Zr nanoparticle surface with LP LenA was further confirmed by zeta potential measurements, which determined a distinct shift from negative ($-12$ mV) to positive zeta potential (+28 mV) after coating (Fig. S6 in the ESM). In the previous work, LP LenA was found to mediate a favorable endosomal release of PMO conjugates, presumably due to endosomal membrane interactions of the contained unsaturated linolenic acid residues. To confirm that the beneficial effects of LP LenA on cellular delivery can also be utilized in combination with PNA-Zr particles, HeLa mRuby3/gal8 reporter cells were used, which can visualize effects on endosomal membrane integrity. The reporter cells express a fusion protein of galectin-8 and mRuby3. Galectin-8 is a cytosolic protein that accumulates at damaged endosomes due to binding to glycosylation moieties located on the inner surface of endosomal membranes^79,80^. HeLa mRuby3/gal8 cells were treated with uncoated 705 SSO-Zr nanoparticles, LP LenA coated 705 SSO-Zr particles or HBG buffer for 8 h. CLSM imaging after additional 4 h incubation with fresh medium showed that HBG buffer and uncoated particles did not lead to a redistribution of cytosolic galectin-8 and no disturbance of endosomal membrane integrity was detectable (Fig. 4C). In contrast, HeLa mRuby3/gal8 cells treated with LP LenA coated particles exhibited a punctuate distribution of mRuby3/gal8 signals with higher fluorescence intensity due to endosomal membrane damage, Overall, the results support the hypothesis that LP LenA can facilitate endosomal escape of PNA-Zr particles.

To determine splice-switching activity, HeLa pluc/705 cells were incubated with 705 SSO-Zr particles or the free PNA-linker with and without LP LenA for 48 h followed by a luciferase activity assay. To rule out any background luciferase activity, luminescence levels were normalized to HBG treated control cells and determined as ‘fold increase in luminescence’^77,78^. As shown in Fig. 4d, an array of different ratios between PNA and LP LenA (1/0.5, 1/0.75, 1/1, 1/1.25, 1/1.5, 1/2, 1/2.5, 1/3) were used.

Cells incubated with 5 $\upmu$M free 705 SSO PNA did not show increased luciferase activity at any PNA to LP LenA mixing ratio. In contrast, covalent conjugation of a DBCO modified 705 SSO PNA derivative to LP LenA via copper-free click chemistry yielded a formulation with distinct splice-switching activity (Fig. S7 in the ESM). Importantly, LP LenA coated 705 SSO-Zr particles clearly increased luminescence levels at the ratios between 1:0.5 to 1:3. The highest increase in luminescence (125-fold) was achieved at a PNA/LP LenA ratio of 1/1.25, which was determined as optimal coating condition. Dose-titrations with free PNA and PNA-Zr nanoparticles at the optimal PNA/LP LenA ratio of 1/1.25 were carried out in a PNA concentration range between 0.156 $\upmu$M to 5 $\upmu$M (Fig. 4e). Consistent with the previous results, free 705 SSO/LP LenA did not induce increased luciferase activity at any concentration, whereas PNA-Zr particles increased luminescence over 20- to 120-fold at PNA concentrations between 0.625 to 5 $\upmu$M. In direct comparison, the LP LenA coated 705 SSO-Zr particles induced competitive levels of luciferase activity as the covalent LP LenA PNA conjugate formulation [Fig. S7 in the ESM]. In contrast, Lipofectamine 3000, which was included as a commercially available transfecting agent, was not able to increase luciferase activity. This observation is not surprising, since PNA represent uncharged nucleic acid analogs and modification with negatively charged or lipophilic moieties would be required for cellular delivery by cationic lipids^81–83^. To additionally confirm the advantage of PNA-Zr nanoparticle formation for cellular delivery, another control experiment was performed (Fig. S8 in the ESM). Phosphate buffer (PBS) was used to compete with PNA linkers and destabilize Zr-linker interactions. 705 SSO-Zr nanoparticles with LP LenA at optimal ratio of 1/1.25 were disassembled by incubation with PBS. Treatment of HeLa pLuc/705 cells with the obtained solution showed negligible effects on luminescence levels. The results demonstrate that the initially very efficient PNA-Zr nanoparticles lost their delivery potential upon particle destruction. In sum, the results demonstrate that bioactive PNA can either be covalently conjugated to carrier molecules or alternatively assembled into nanoparticles for cellular delivery.

To assess the kinetic of splice-switching and onset of luciferase expression, the impact of incubation time was investigated with 705 SSO-Zr/LP LenA particles at the optimal coating ratio (1/1.25). HeLa pLuc/705 cells were incubated with varied doses of PNA-Zr nanoparticles for 12, 24, 36 and 48 h and the luciferase activity was determined (Fig. S9 in the ESM). The reason for the apparent lag in luciferase induction is not obvious, but it could be related to slow release of the PNA from endosomes, slow release of the PNA from the nanoparticles and the essential intracellular trafficking into the nucleus.

To confirm that the increased luciferase activity is indeed related to sequence specific effects of the splice-switching antisense PNA, particles composed of PNA linkers with alternative sequences (PAMBA-GAGTATGAGA-TPA and PAMBA-GCAGCT-TPA) were used as controls (Fig. S10 in the ESM). HeLa pLuc/705 cells were treated with the control PNA-Zr particles with LP LenA coating at optimal 1/1.25 ratio for 48 h. The subsequent luciferase activity assay determined that particles without 705 SSO PNA did not increase luciferase activity, which confirms that the observed effect of 705 SSO particles are sequence dependent.

The fact that particles can be disassembled by excessive amounts of phosphate ions raised the question about the stability at different physiological phosphate concentrations (e.g. 1.12 mM to 1.45 mM in serum)^84^. To address this aspect particle stability was measured in different PBS dilutions leading to varied phosphate concentrations, as well as in 20% filtered (0.2 $\upmu$m) fetal bovine serum (FBS) (Fig. S11 in the ESM). Here, a phosphate concentration dependent decrease of particle stability was observed, which could lead to decreased biological stability but also a favorable intracellular release of PNA, since the phosphate concentration is higher inside cells. Similarly, the particles showed a sensitivity towards the presence of FBS and dissociated over the course of 1 h. Notably, the coating with LP LenA clearly increased the serum stability of the particles, thereby providing another advantage apart from facilitating endosomal escape.

The intended splice-switch of mutated $\beta$-globin intron in the HeLa pLuc/705 model was additionally confirmed by RT-PCR (Fig. 4f, Fig. S12 in the ESM). Total RNA was extracted from HeLa pLuc/705 cells 48 h after the treatment with 705 SSO-Zr/LP LenA at optimal 1/1.25 ratio. Afterwards, the sequence surrounding $\beta$-globin IVS2 was amplified by RT-PCR and analyzed via agarose electrophoresis. The single band (268 bp) obtained from HBG treated control cells represents the unchanged aberrant splicing product. In case of 705 SSO-Zr nanoparticle treated cells, a dose-dependent appearance of an additional band (142 bp) corresponding to the splice-corrected product was seen. In contrast to the luciferase measurements, this assay is (semi)quantitative, thus indicating about 22%, 9% and 5 % splice correction at 5 $\upmu$M, 2.5 $\upmu$M and 1.25 $\upmu$M PNA concentration, respectively (determined by image data analysis of band intensities).

Potential cytotoxicity of the PNA-Zr particles was investigated by CellTiter-Glo$^\circledR$ assay with HeLa wild type cells (Fig. S7(b), S13 in the ESM). Analogously to the splice-switching experiments, cells were incubated for 48 h with free PNA or PNA-Zr particles at different concentrations and LP LenA ratios. Without LP LenA, neither free PNA, nor PNA-Zr particles mediated effects on metabolic activity at 5 $\upmu$M, whereas in combination with the lipopeptide a dose- and ratio-dependent reduction of metabolic activity was evident above 1.25 $\upmu$M and pronounced at 5 $\upmu$M (Fig. S10(b), S13 in the ESM). Concordantly, lower cellular luciferase activity was observed in cells treated with 5 $\upmu$M PNA-Zr-LP LenA particles compared to 2.5 $\upmu$M (Fig. 4e), which is consistent with a lower cell viability. It is not surprising that LP LenA, in analogy to other potent delivery agents and cationic lipids, affects cell viability in a dose-dependent manner, e.g. by disturbing lipid membrane integrity^85,86^. Nevertheless, the combination of PNA-Zr particles with LP LenA coating is considered a feasible strategy to increase particle stability, facilitate endosomal release and enhance intracellular PNA delivery. Indeed, optimization of particle parameters, coating ratios and composition of the coating agent may lead to a favorable balance between delivery efficiency and tolerability.

## Conclusions

This work reports novel metal-organic nanoparticles, generated via coordination driven self-assembly of peptide nucleic acid linkers and Zr(IV) nodes. A systematic variation of PNA linker architectures identified essential design and synthesis parameters: (1) single- and double-stranded PNA linkers require two aromatic carboxylic acid functions (PAMBA, TPA) for the coordinative interaction with Zr(IV) nodes; (2) double-stranded PNA linkers with separated aromatic carboxylic acids on both strands require at least 6-mer sequences; (3) nanoparticle synthesis requires a two-step procedure with preformation of $Zr_{6}O_{4}(OH)_{4}$ nodes, before assembly with PNA linkers at RT; (4) nanoparticles built from double-stranded PNA linkers with separated aromatic carboxylic acids on both strands are sensitive towards thermal degradation, presumably due to PNA linker melting; and (5) nanoparticles built from PNA linkers with both aromatic carboxylic acids on a single strand exhibit the highest stability. The amorphous PNA-Zr nanoparticles feature an extraordinary PNA loading capacity of > 94% w/w, are readily taken up by cells and can deliver functional antisense PNAs after coating with a cationic lipopeptide to increase particle stability and facilitate endosomal release. PNA-Zr nanoparticles are sensitive towards competing interactions and degrade over time upon exposure to phosphate ions or serum; finally, successful modulation of mRNA splicing was achieved by delivering a splice-switching antisense PNA via lipopeptide coated PNA-Zr nanoparticles.

Based on the identified design criteria, a highly flexible platform has been established, which combines customizable PNA linker chemistry with coordinative driven self-assembly to generate nanoparticles with imprinted biological activity. It is suggested that the presented PNA-Zr nanoparticles platform provides high flexibility for the generation of sequence customizable, bioactive nanoparticles with possible potential for drug formulation in RNA therapeutics.

## Methods

### Materials

All reagents were purchased from commercial chemical suppliers. Reagents were used as received without further purification unless otherwise stated. The reagents used for the experiments are summarized in Table S1. Buffers used for the experiments are summarized with their composition in Table S2.

### Resin loading with 4-(aminomethyl)benzoic acid (PAMBA)

An amount of 1 g of 2-chlorotrityl chloride resin (approx. 1.6 mmol chloride) was pre-swelled for 30 min in 10 mL of dry dichloromethane (DCM) in a syringe reactor (Multisyntech, Witten, Germany). Then DCM was removed by vacuum filtration. The resin was agitated for 60 min with a solution containing 3 mL of *N,N*-dimethylformamide (DMF), 4 mL of dry DCM, 0.6 mmol of 4-(Fmoc-aminomethyl)benzoic acid (Fmoc-PAMBA-OH) and 1.8 mmol *N,N*-diisopropylethylamine (DIPEA). Afterwards, the solution was discarded by vacuum filtration and a capping solution containing 4 mL DCM, 3 mL methanol and 500 $\upmu$L DIPEA was added. After agitation for 30 min, the solution was removed and the resin was washed three times with DMF and DCM. The resin was dried under high vacuum and three samples were taken for loading determination. The samples were agitated with 1 mL of 20% piperidine in DMF at RT for 1 h. 50 $\upmu$L of the supernatant of each sample was diluted with 1.95 mL DMF and the absorbance at 301 nm was measured with a Cary 3500 UV-Vis spectrophotometer (Agilent Technologies, U.S.). As blank solution, 50 $\upmu$L of 20% piperidine in DMF diluted with 1.95 mL DMF was used. The resin loading was determined based on the following formula:

$$\hbox {resin load} [\hbox{mmol}/\hbox{g}]\,=\,(A_{301}\,\textrm{nm}\times 1000)/(\hbox {resin mass} [\hbox{mg}]\times 7800\,[\hbox {L}\times \,\textrm{mol}^{-1}\,\times \,\textrm{cm}^{-1}]\times 0.025)$$

### Solid phase synthesis

PNA syntheses were carried out with a Biotage Initiator+ SP Wave semiautomatic peptide synthesizer (Biotage, Uppsala, Sweden) at 30 $\upmu$mol scales in 10 mL reactors. For the synthesis of PNA sequences with C-terminal PAMBA, PAMBA loaded 2-chlorotrityl chloride resin, otherwise a H-Rink-Amide-Chemmatrix® resin was used. Initially, the required amount of resin was pre-swelled in 5 mL of DCM for 30 min. Then, the resin was deprotected by four times incubation with 3 mL of 20% piperidine in *N*-methyl-2-pyrrolidone (NMP) for 10 min at RT, followed by five washing steps with 4 mL NMP. Coupling of PNA monomers was carried out by incubating 4 eq. of PNA monomer, 4 eq. of Oxyma and 4 eq. of DIC with the resin for 6 min at $75\,^{\circ }\hbox {C}$ (microwave irradiation). Each coupling step was followed by a threefold resin wash with NMP and an acetylation reaction to block residual free amines. Acetylation was carried out by 3 min incubation at RT with 2.25 mL of a mixture containing NMP/2,6-lutidine/acetic anhydride at the ratio 89/6/5 (v/v/v). Fmoc deprotection steps were carried out by four times incubation with 2.4 mL of 20% piperidine in NMP at RT for 10 min, followed by five times resin wash with 2.5 mL NMP. After each coupling and deprotection step, a Kaiser test was carried out to determine presence of free amines. For this, a small resin sample was transferred into a 1.5 mL reaction tube and one drop of each 80% phenol in ethanol (w/v), 5% ninhydrin in ethanol (w/v) and 20 $\upmu$M potassium cyanide in pyridine were added. The mixture was incubated at $99\,^{\circ }\hbox {C}$ for 4 min under shaking. The Kaiser test indicates presence of free amines by change of the yellow solution to a deep blue color, otherwise the color does not change. After the PNA sequence has been completed, N-terminal modification with TPA was accomplished with a slightly modified coupling procedure. TPA was coupled by incubation of the resin with a mixture containing 4 eq. of mono-*tert*-butyl terephthalate, 4 eq. 1-hydroxybenzotriazole (HOBt), 4 eq. benzotriazol-1-yloxy-tripyrrolidinophosphonium hexafluorophosphate (PyBOP) and 8 eq. DIPEA dissolved in DMF for 10 min at $70\,^{\circ }\hbox {C}$ (microwave irradiation). N-terminal acetic acid was introduced by an acetylation step as described above. Finally, the resin was washed three times with DMF, three times with DCM. The resin was dried under vacuum and stored in an airtight container at $4\,^{\circ }\hbox {C}$ until cleavage and work-up of the PNA construct.

### Cleavage and purification of the PNA product

PNA products were cleaved from previously dried resin by adding a cleavage cocktail containing 2.85 mL trifluoroacetic acid (TFA), 75 $\upmu$L triisopropylsilane (TIS) and 75 $\upmu$L water. The resin was agitated for 90 min with cleavage cocktail. The cleavage solution was added into 45 mL of a pre-cooled mixture of methyl-*tert*-butyl ether (MTBE) and *n*-hexane 1:1 (v/v) in a 50 mL reaction tube, which was centrifuged for 5 min at 4000 rpm (Megafuge 1.0 R, Heraeus, Hanau, Germany). The supernatant was removed and the PNA pellet dried under a nitrogen flow. The dried product was dissolved in 30% acetonitrile in water and snap-frozen in liquid nitrogen. Freeze drying was performed with a Christ Alpha 2–4 LD plus (Martin Christ Gefriertrocknungsanlagen GmbH, Osterode, Germany). The product was purified by size exclusion chromatography using a Sephadex G-10 column connected to an Äkta purifier system (GE Healthcare Bio-Sciences AB, Uppsala, Sweden) and 30% acetonitrile in water as mobile phase. The average yield of PNA synthesis was around 60%.

### MALDI mass spectrometry

Samples were spotted on a MTP AnchorChip (Bruker Daltonics, Bremen, Germany) by first crystallizing 1 $\upmu$L of saturated Super-DHB matrix solution (2,5-dihydroxybenzoic acid and 2-hydroxy-5-methoxybenzoic acid), followed by addition of 1 $\upmu$L of sample solution. After drying, mass spectra were recorded in positive mode with a Autoflex II mass spectrometer (Bruker Daltonics, Bremen, Germany).

### Particle synthesis

PNA-Zr particle synthesis required a two-step process to enable the assembly at RT and avoid melting of PNA duplexes. A previously reported method for the synthesis of UiO-66 at RT served as basis with adaption^66^. First step was the formation of Zr nodes, which was achieved by mixing 71 $\upmu$L of a 70 wt.% zirconium(IV) propoxide solution in 1-propanol (0.0519 g, 0.158 mmol), 7 mL of DMF, and 4 mL of acetic acid in a sealed glass container. The solution was heated to $130\,^{\circ }\hbox {C}$ for 2 h. Then, a noticeable change in color from colorless to yellow was observed. The solution was allowed to cool to RT before continuation with the second step. Double-stranded PNA linkers were subjected to an annealing process, by combining the two complementary strands at equimolar ratio (only 1 strand in case of palindromic sequences) in aqueous solution, heating to $90\,^{\circ }\hbox {C}$ over 30 min and cooling to RT over 30 min. Then, 30% acetonitrile was added, samples were snap-frozen in liquid nitrogen and freeze-dried. PNA linkers were dissolved in DMF at concentrations of 1-4 mg/mL, depending on the solubility. Finally, the pre-formed Zr node solution was mixed with the PNA linker at equimolar ratio. The mixture was incubated for 24 h at RT under stirring. The formed PNA-Zr particles were separated from the solution by centrifugation for 5 min at 4000 rpm and were washed three times by repeated resuspension in DMF and centrifugation. Afterwards, the particles were washed twice with ethanol, resuspended in 1 mL ethanol and stored at $4\,^{\circ }\hbox {C}$.

### PNA encapsulation efficiency

The PNA encapsulation efficiency (EE) in Zr nanoparticles was determined by quantifying the molecular amount of PNA via UV absorption at 260nm (164.3 mL/($\upmu$mole $\times$ cm) extinction coefficient for 705 SSO PNA). After PNA-Zr nanoparticle formation, particles were decomposed by phosphate competition and the initial amount of released PNA was normalized to the amount of PNA used for the synthesis according to the following formula:

$$\begin{aligned} \hbox {EE }[\%]\,=\,(PNA_{release}\,\hbox {[nmol]}\,/\,PNA_{feed}\,\hbox {[nmol]}) \times 100 \% \end{aligned}$$

### X-ray diffraction

An amount of approx. 2 mg dry PNA-Zr particles was used for XRD measurement with a STOE transmission diffractometer system Stadi MP with Cu K$\alpha$1 radiation ($\lambda$ = 1.54060 Å) and a Ge (111) single crystal monochromator. Diffraction patterns were recorded with a DECTRIS solid-state strip detector MYTHEN 1K (step size of $4.71\,^{\circ }$, counting time of 120 s per step). Generated data was analyzed with WinXPOW RawDat v3.0.2.5.

### Nitrogen sorption measurements

Approximately 20 mg of dry PNA-Zr particles were degassed at RT under high vacuum for 38 h. Nitrogen sorption isotherm were measured with an Autosorb iQ Station 1 at $-195.8\,^{\circ }\hbox {C}$ and the generated data was evaluated using the ASiQwinTM software (Version 3.0, Quantachrome Instruments). Surface area of the samples were calculated by linearized Brunauer–Emmett–Teller (BET) equation. Pore sizes were calculated according to quenched solid density functional theory carbon model (at $-195.8\,^{\circ }\hbox {C}$, nitrogen and relative pressure between 0 to 1 atm).

### ICP-AES

Inductively coupled plasma atomic emission spectroscopy (ICP-AES) was performed with three independent samples. Samples were first vacuum dried for 24 h followed by 4 h drying at $90\,^{\circ }\hbox {C}$. Digestion was performed with 69% HNO3 in water until complete dissolution. Then the samples were diluted with 3% HNO3 in double distilled water and the Zr content was measured by ICP-AES (CCD simultaneous ICP AES Vista RL by Agilent, wavelengths 257.2, 327.3, 339.2, 343.8 and 349.7, suction time 35 s, stabilization time 45 s, power 1.25 kW). The average of the Zr to total mass ratio of three samples was calculated.

### DLS and zeta potential

Size and zeta potential measurements were performed with a Zetasizer Nano ZS with backscatter detection (Malvern Instruments, Worcestershire, UK) using DTS1070 folded capillary cuvettes. PNA-Zr particles were sonicated for 5 min before the measurements. For size and PDI determinations, approximately 2.5 $\upmu$g of PNA-Zr particles was added to 125 $\upmu$L of distilled water. The refractive index was set to 1.330 and the viscosity to 0.8872 cP. Measurements were carried out at RT with 1 min equilibration time and at least 6 subruns. Afterwards, 835 $\upmu$L 10 mM NaCl was added to the samples for zeta potential determination by ELS. Samples were measured three times at RT with 1 min equilibration time and at least 12 subruns. Zeta potentials were automatically calculated based on the Smoluchowski equation.

### SEM

Stock solutions of PNA-Zr particles were vortexed and diluted 1:10 with ethanol. The diluted solutions were spotted onto the hydrophobic surface of a SEM sample holder. Dried samples were coated with a carbon layer by three cycles of carbon vacuum deposition. SEM measurements were carried out with a Dual beam FEI Helios G3 UC SEM operated at 3 kV.

### Particle stability

The stability of PNA-Zr particles in presence of HBG and HEPES was investigated by photometric measurement of the optical density of particle suspensions. For this purpose, approximately 50 $\upmu$L of PNA-Zr stock solutions in ethanol was added into quartz cuvettes with 2 mL of HBG or HEPES. The same volume of ethanol was added into separate cuvettes with HBG or HEPES as blank controls. Absorbance of the samples at 400 nm was monitored in 1 min intervals over 1 h. Data was collected with a Cary 3500 UV-Vis spectrophotometer (Agilent Technologies, U.S.) under constant stirring. For thermal stability assessments, PNA-Zr particles were added into 2 mL water. The same volume of ethanol was added into separate cuvettes with water as blank control. Temperature probes were placed into the cuvettes and the samples were exposed to a temperature gradient between $25\,^{\circ }\hbox {C}$ to $70\,^{\circ }\hbox {C}$ with an increase of $1\,^{\circ }\hbox {C}$ per min. Absorbance of the samples at 400 nm was measured after each $0.5\,^{\circ }\hbox {C}$ temperature increase. Data was collected with a Cary 3500 UV-Vis spectrophotometer (Agilent Technologies, U.S.) under constant stirring. Particle stability in different dilutions of PBS and in 20 % FBS was investigated similarly by photometric measurement of the optical density of particle suspensions. Different PBS dilutions were prepared and approximately 50 $\upmu$L of PNA-Zr particle suspension was added into quartz cuvettes to 2 mL of solution. The same volume of ethanol was added into separate cuvettes with PBS dilution as blank control. For the stability determination in presence of serum, 20% FBS in water was prepared and 50 $\upmu$L of PNA-Zr particle suspension was added into 2 mL of 20% FBS. As blank 2 mL 20% FBS with 50 $\upmu$L of ethanol was used. Absorbance values of the samples at 400 nm wavelength were determined each min for 1 h. Data collection was performed automatically with a Cary 3500 UV-Vis spectrophotometer (Agilent Technologies, U.S.) under continuous stirring.

### Cell culture

HeLa pluc/705 and HeLa wild type cells were cultured in low glucose (1 g/L glucose) Dulbecco’s Modified Eagle Medium (DMEM). DMEM was supplemented with 10% FBS, 100 U/mL penicillin and 100 $\upmu$g/mL streptomycin. The cells were cultivated in ventilated flasks in the incubator at $37\,^{\circ }\hbox {C}$ and 5% CO_2_ in a humidified atmosphere.

### Particle coating

The artificial lipopeptide LP LenA, which was previously used for conjugation with splice-switching PMOs^78^, was used for coating of PNA-Zr nanoparticles. LP LenA was synthesized by solid-phase synthesis and has the sequence K_ε_(N_3_)-Y_3_-Stp_2_-K_ε_[G-K_α,ε_(linolenic acid)_2_]-Stp_2_-Y_3_ (Y: tyrosine; Stp: succinyl-tetraethylenepentamine; K: lysine; G: glycine; K_ε_(N_3_): azidolysine). The 705 SSO PNA content of nanoparticles was determined by disassembly of a small sample via competition with PBS and photometric quantification of released PNA at 260 nm (164.3 ml/($\upmu$mole $\times$ cm) extinction coefficient). Based on the determined PNA content, the nanoparticles were incubated with stoichiometric amounts of lipopeptide LP LenA for 40 min before further dilution in HBG.

### PNA LP LenA conjugate formation

PNA with the sequence H-CCTCTTACCTCAGTTACA-NH$_{2}$ (1 eq.) was dissolved in 500 $\upmu$L water free DMSO with 10 equivalents of DIPEA. DBCO-NHS (5 eq.) in 250 $\upmu$L of water free DMSO was added to the PNA solution and incubated overnight at RT. Afterwards, the sample was purified by size exclusion chromatography using an Äkta purifier system (GE Healthcare Bio-Sciences AB, Uppsala, Sweden), a Sephadex G-10 column and 30% acetonitrile in water as mobile phase. The pooled product fractions were freeze dried and stored at $\hbox {-}20\,^{\circ }\hbox {C}$. One day before the cell experiment, a 100 $\upmu$M PNA-DBCO solution in HBG supplemented with 0.04 M HCl and a 300 $\upmu$M LP LenA solution in HBG were prepared. Equal volumes of both solutions were combined and incubated over night at RT with shaking at 300 rpm. For cell experiments, the PNA conjugate formulation was diluted with HBG to the desired concentrations.

### PNA Lipofectamine 3000 formulation

The protocol by the manufacturer for plasmid DNA (pDNA) transfections with Lipofectamine 3000 (ThermoFisher Scientific, USA) was adapted to PNA treatments. The required amount of Lipofectamine 3000 and P3000 for 200 ng pDNA per well was used. With fixed Lipofectamine 3000 amount, the amount of 705 SSO PNA was varied to obtain transfection solutions of 50 $\upmu$M, 25 $\upmu$M, 12.5 $\upmu$M, 6.25 $\upmu$M, 3.125 $\upmu$M, and 1.5625 $\upmu$M. P3000 and the required amount of 705 SSO PNA were combined in 25 $\upmu$L of serum-free DMEM. 1 $\upmu$L Lipofectamine 3000 was diluted in 25 $\upmu$L serum-free DMEM and combined with the solution containing P3000 and PNA. The mixed solution was incubated at RT for 15 min.

### Luciferase activity assay

HeLa pluc/705 cells were seeded in 96-well plates at a density of $5\times 10^{3}$ cells per well 24 h prior to the treatments. The cell treatment was initiated by replacement of the medium with 90 $\upmu$L fresh serum-containing DMEM and 10 $\upmu$L of PNA formulations was added to each well. After 48 h incubation (or alternative incubation times as stated in kinetic studies), the medium was removed and 100 $\upmu$L of lysis buffer (Promega, Mannheim, Germany) was added to each well. Cells were incubated with lysis buffer for 45 min at RT. Luciferase activity in 35 $\upmu$L of cell lysate was measured with luciferin-LAR buffer solution (1 mM luciferin, 20.94 mM glycylglycine, 1 mM MgCl2, 0.1 mM EDTA, 3.29 mM DTT, 0.55 mM ATP, 0.28 mM Coenzyme A stock solution, pH 8.0-8.5) using a Centro LB 960 plate reader luminometer (Berthold Technologies, Bad Wildbad, Germany). The relative light units (RLU) were normalized to HBG-treated cells and the results are presented as ‘fold increase in luminescence’. All experiments were carried out in triplicates.

### Metabolic activity

Cell viability of HeLa wild type cells was investigated via CellTiter-Glo$^\circledR$ assay after treatment with PNA-Zr particles. HeLa cells were seeded in 96-well plates at a density of $5 \times 10^{3}$ cells per well 24 h prior to the treatments. Then, the medium was replaced with 90 $\upmu$L fresh serum-containing DMEM and 10 $\upmu$L of PNA-Zr particles was added to each well. After 48 h, the medium was removed and 25 $\upmu$L of DMEM and 25 $\upmu$L of CellTiter-Glo$^\circledR$ Reagent (Promega, Mannheim, Germany) was added to each well. The well plates were incubated for 30 min at RT under shaking and bioluminescence was measured using a Centro LB 960 plate reader luminometer (Berthold Technologies, Bad Wildbad, Germany). All experiments were carried out in triplicates. Relative metabolic activities were calculated by normalization to HBG-treated control wells, according to the following formula:

$$\begin{aligned} \hbox {cell viability }[\%]\,=\,\hbox {mean(sample) }/\hbox { mean(control) }\times \,100\,\% \end{aligned}$$

### RT-PCR

HeLa pluc/705 cells were seeded in 24-well plates at a density of $5 \times 10^{4}$ cells per well 24 h prior to the treatments. Then, the medium was replaced with 900 $\upmu$L fresh serum-containing DMEM and 100 $\upmu$L of particle suspensions with varied concentrations was added to each well. Cells were incubated for 48 h, then the medium was removed and total RNA isolation was performed with Tri-Reagent$^\circledR$ (Sigma-Aldrich) according to the manufacturer’s protocol. A High-Capacity cDNA Reverse Transcription Kit (Applied Biosystems) was used according to the manufacturer’s protocol to reverse-transcribe the isolated RNA into cDNA. Standard PCR was carried out with 300 ng of cDNA using the HotStarTaq Plus DNA polymerase kit (QIAGEN) and the following primers: 5$^{\prime }$-TTGATATGTGGATTTCGAGTCGTC-3$^{\prime }$ (forward) and 5$^{\prime }$-TGTCAATCAGAGTGCTTTTGGCG-3$^{\prime }$ (reverse). PCR program was composed of an initial cycle at $95\,^{\circ }\hbox {C}$ for 5 min, followed by 29 cycles with $95\,^{\circ }\hbox {C}$ for 30 s, $55\,^{\circ }\hbox {C}$ for 30 s, $72\,^{\circ }\hbox {C}$ for 30 s and finally one cycle at $72\,^{\circ }\hbox {C}$ for 10 min. The RT-PCR product was analyzed via agarose gel electrophoresis (1.25% agarose in TBE buffer containing GelRed$^\circledR$) at 90 V for 45 min.

### Calcein loading to PNA-Zr particles

PNA-Zr particle suspensions in ethanol were centrifuged and the supernatants were removed. The particles were resuspended in 1 mL of 0.25 mM calcein solution in water and incubated on a shaker for 15 min at RT. Afterwards, the particles were centrifuged and washed three times with water, three times with ethanol and stored in ethanol. 50 to 100 $\upmu$L of calcein labeled PNA-Zr suspensions were disassembled via competition with 1 mL PBS. The PNA and calcein content was quantified photometrically with a Cary 3500 UV-Vis Spectrophotometer at 260 nm (PNA) and 515 nm (calcein), respectively. For PNA-Zr particles based on GCATGC-TPA a PNA:calcein ratio of 100:1.22 (w/w), based on PAMBA-GCAGCT-TPA a ratio of 100:1.32 (w/w) was determined.

### Confocal laser scanning microscopy (CLSM)

To investigate cellular uptake of calcein loaded particles, HeLa pLuc/705 cells were seeded in 8 well-Ibidi $\upmu$-slides (Ibidi GmbH, Planegg/Martinsried, Germany) at a density of $2 \times 10^{4}$ cells per well 24 h prior to the treatments. Then, the medium was replaced with 240 $\upmu$L fresh serum-containing DMEM and 60 $\upmu$L of calcein labeled PNA-Zr particles. Cells were incubated for 4 h with particles, then the medium was replaced with fresh medium and incubation was continued for 4 h. Cells were washed with PBS and fixated with 300 $\upmu$L/well 4% paraformaldehyde for 45 min at RT. Each well was washed twice with PBS and the slide was stored in PBS at $4\,^{\circ }\hbox {C}$ overnight. Cell staining was performed by incubation with 300 $\upmu$L PBS containing DAPI (2 $\upmu$g/mL) for staining of nuclei and phalloidine-rhodamine (1 $\upmu$g/mL) for staining of actin cytoskeleton for 30 min at RT in a light-protected environment. The staining solution was removed and replaced with 300 $\upmu$L PBS.

To assess the effect of LP LenA coated particles on endosomal membrane integrity, HeLa mRuby3/gal8 reporter cells were used,^87^ which are based on the construct PB-CAG-mRuby3-Gal8-P2A-Zeo provided by Jordan Green (Addgene plasmid # 150815; http://n2t.net/addgene:150815; RRID:Addgene_150815). For the assessment, $2\times 10^{4}$ HeLa mRuby3/gal8 cells in 300 $\upmu$L DMEM medium were seeded in 8 well-Ibidi $\mu$-slides. Cells were incubated at $37\,^{\circ }\hbox {C}$ and 5% CO_2_ for 24 h. Then medium was removed and 270 $\upmu$L fresh medium and 30 $\upmu$L of LP LenA coated (1/1.25 molar ratio) or uncoated 705 SSO-Zr particle suspensions containing 25 $\upmu$M PNA were added to each well. Cells were incubated for 8 h with particles then the solutions were removed and incubation was continued with fresh medium for 4 h. Then, cells were washed with PBS and each well was fixated with 300 $\upmu$L 4% paraformaldehyde for 45 min at RT. After fixation, wells were washed again twice with PBS and stored in PBS at $4\,^{\circ }\hbox {C}$. Staining of nuclei was performed by incubation for 30 min (at RT in light protected environment) with 300 $\upmu$L PBS solution containing DAPI (2 $\upmu$g/mL). Staining solution was removed and replaced with 300 $\upmu$L PBS. Images were taken with a Leica-TCS-SP8 confocal laser scanning microscope equipped with a HC PL APO 63x 1.4 objective (Germany) and LAS X software from Leica. DAPI emission was recorded at 460 nm, calcein at 530 nm, Phalloidine-Rhodamine at 580 nm and mRuby3 at 585 nm.

### Statistical analysis

Results are presented as arithmetic mean ± standard deviation. Significance levels were evaluated by two-tailed t-test (unpaired). The following symbols are used to indicate significance levels: *p $\le$ 0.05, **p $\le$ 0.01, ***p $\le$ 0.001, ****p $\le$ 0.0001 and ‘ns’ for not significant.

### Supplementary Information

Supplementary Information.

## Data Availability

The datasets used and/or analysed during the current study are available from the corresponding author on reasonable request.
