# Supplementary material for: Peptide nucleic acid-zirconium coordination nanoparticles
Source: Sci Rep. 2023 Aug 30;13:14222. doi: 10.1038/s41598-023-40916-w (PMC10469198; doi:10.1038/s41598-023-40916-w)
Supplement: Supplementary file 1 — Supplementary Information. [file 41598_2023_40916_MOESM1_ESM.pdf]

Supplementary Information

Peptide Nucleic Acid – Zirconium Coordination Nanoparticles

Özgür Öztürk,[1,2] Anna-Lina Lessl,[1] Miriam Höhn,[1] Stefan Wuttke,[3,4] Peter E. Nielsen,[5] Ernst Wagner[1] and Ulrich Lächelt[1,6,*]

[1] Department of Pharmacy and Center for NanoScience (CeNS), LMU Munich, 81377 Munich, Germany

[2] Department of Genetic and Bio Engineering, Alanya Alaaddin Keykubat University, Antalya, Türkiye

[3] Basque Center for Materials (BCMaterials), Leioa, Spain

[4] Ikerbasque, Basque Foundation for Science, Bilbao, Spain

[5] Department of Cellular and Molecular Medicine, University of Copenhagen, Copenhagen, Denmark

[6] Department of Pharmaceutical Sciences, University of Vienna, Austria

* Corresponding author: Ulrich Lächelt, E-Mail: ulrich.laechelt@univie.ac.at

Table of Contents

1. Materials.......... 2

2. Supporting Figures and Tables 4

3. Analytical data 11

4. References 18

1. Materials

All reagents were purchased from commercial chemical suppliers. Reagents were used as received without further purification unless otherwise stated. The reagents used for the experiments are summarized in Table S1. Buffers used for the experiments are summarized with their composition in Table S2.

PNA syntheses were carried out with a Biotage Initiator+ SP Wave semiautomatic peptide synthesizer (Biotage, Uppsala, Sweden). Disposable syringe microreactors were purchased from Multisyntech (Witten, Germany) with pre-fitted polytetrafluoroethylene (PTFE) filters.

Table S1 Reagents used for experimental procedures

Materials	CAS-No./Cat-No.	Supplier
**1-Hydroxybenzotriazole hydrate**	123333-53-9	Sigma-Aldrich (Munich, Germany)
**2,6-Lutidine**	108-48-5	Sigma-Aldrich (Munich, Germany)
**2-Chlorotritylchloride resin**	42074-68-0	Iris Biotech (Marktredewitz, Germany)
**4-(Fmoc-aminomethyl)benzoic acid**	164470-64-8	Iris Biotech (Marktredewitz, Germany)
**4-(tert-Butoxycarbonyl)benzoic acid**	20576-82-3	BLD Pharmatech GmbH (Mehlingen Germany)
**4',6-diamidino-2-phenylindole (DAPI)**	D9542	Sigma-Aldrich (Munich, Germany)
**Acetic anhydride**	108-24-7	Sigma-Aldrich (Munich, Germany)
**Acetonitrile**	75-05-8	VWR Int. (Darmstadt, Germany)
**Agarose NEEO Ultra**	9012-36-6	Carl Roth (Karlsruhe, Germany)
**Beetle luciferin sodium salt**	E1605	Promega (Mannheim, Germany)
**Calcein**	154071	Sigma-Aldrich (Munich, Germany)
**CellTiter-Glo®**	G7571/2/3	Promega (Mannheim, Germany)
**Dichloromethane**	75-09-2	Bernd Kraft (Duisburg, Germany)
**DMEM**	D6046	Sigma-Aldrich (Munich, Germany)
**Ethanol absolute**	64-17-5	VWR Int. (Darmstadt, Germany)
**Fetal bovine serum (FBS)**	F9665	Sigma-Aldrich (Munich, Germany)
**Flasks and multi-well plates**	-	TPP (Trasadingen, Switzerland)
**Fmoc-PNA-A(Bhoc)-OH**	186046-82-2	ASM Research Chemicals GmbH (Hannover, Germany)
**Fmoc-PNA-C(Bhoc)-OH**	186046-81-1	ASM Research Chemicals GmbH (Hannover, Germany)
**Fmoc-PNA-G(Bhoc)-OH**	186046-83-3	ASM Research Chemicals GmbH (Hannover, Germany)
**Fmoc-PNA-T-OH**	169396-92-3	ASM Research Chemicals GmbH (Hannover, Germany)
**GelRed**	41003	VWR International GmbH (Darmstadt, Germany)
**HEPES**	7365-45-9	Biomol (Hamburg, Germany)
**H-Rink-Amide-ChemMatrix®**	CM-7600	Iris Biotech (Marktredewitz, Germany)

**Lipofectamine 3000**	L3000001	Thermo Fisher Scientific, USA
**LP LenA**	-	In house synthesis (cf. Kuhn et al.)
**Luciferase cell culture lysis buffer**	E1500	Promega (Mannheim, Germany)
**Methanol**	67-56-1	Fisher Scientific (Schwerte, Germany)
**Methyl-tert-butyl ether**	1634-04-4	Brenntag (Mülheim/Ruhr, Germany)
***N,N'*-Diisopropylcarbodiimide**	693-13-0	Iris Biotech (Marktredewitz, Germany)
***N,N*-Diisopropylethylamine**	7087-68-5	Iris Biotech (Marktredewitz, Germany)
***N,N*-Dimethylformamide**	68-12-2	Iris Biotech (Marktredewitz, Germany)
***n*-Hexane**	110-54-3	Brenntag (Mülheim/Ruhr, Germany)
***N*-Methyl-2-pyrrolidone**	872-50-4	Iris Biotech (Marktredewitz, Germany)
**OxymaPure**	3849-21-6	Iris Biotech (Marktredewitz, Germany)
**pCMVLuc**	PF461	PlasmidFactory (Bielefeld, Germany)
**Penicillin-Streptomycin**	P4333	Sigma-Aldrich (Munich, Germany)
**Piperidine**	110-89-4	Iris Biotech (Marktredewitz, Germany)
**Pybop®**	128625-52-5	Merck KGaA (Darmstadt, Germany)
**Rhodamine-Phalloidin**	R415	Life Technologies/Thermo Fisher Scientific (Munich, Germany)
**Sephadex® G-10**	9050-68-4	GE Healthcare (Freiburg, Germany)
**Terephthalic acid**	100-21-0	Sigma-Aldrich (Munich, Germany)
**Trifluoroacetic acid**	76-05-1	Iris Biotech (Marktredewitz, Germany)
**Triisopropylsilane**	6485-79-6	Sigma-Aldrich (Munich, Germany)
**Trypsin-EDTA** - (10x) Trypsin 0.5 %/EDTA 0.2 % in PBS, w/o: Ca and Mg	P10-024100	PAN-Biotech (Aidenbach, Germany)
**Zirconium(iv) propoxide solution**	23519-77-9	Sigma-Aldrich (Munich, Germany)

Table S2 Buffers used for experimental procedures

**Buffer**	**Composition**
**Freeze-dry solvent**	30% (v/v) acetonitrile in water
**HBG**	20 mM HEPES, 5 % glucose, pH 7.4
**HEPES**	20 mM HEPES, pH 7.4
**LAR Buffer**	20 mM glycylglycine, 1 mM $MgCl_2$, 0.1 mM EDTA, 3.29 mM DTT, 0.548 mM ATP (adenosine 5'-triphosphate), 0.55 mM Coenzyme A stock solution, pH 8.0-8.5
**Luciferin**	10 mM Luciferin-Na, 1M Glycylclycine, pH 8.0,
**Phosphate-buffered saline (PBS)**	137 mM NaCl, 2.7 mM KCl, 8.1 mM Na_2HPO_4, 1.5 mM KH_2PO_4, pH 7.3-7.5
**Size Exclusion Chromatography Mobile Phase**	700 mL water, 300 mL acetonitrile
**TBE buffer**	89 mM Trizma® base, 89 mM boric acid, 2 mM EDTA-$Na_{2,}$ pH 8.0

2. Supporting Figures and Tables

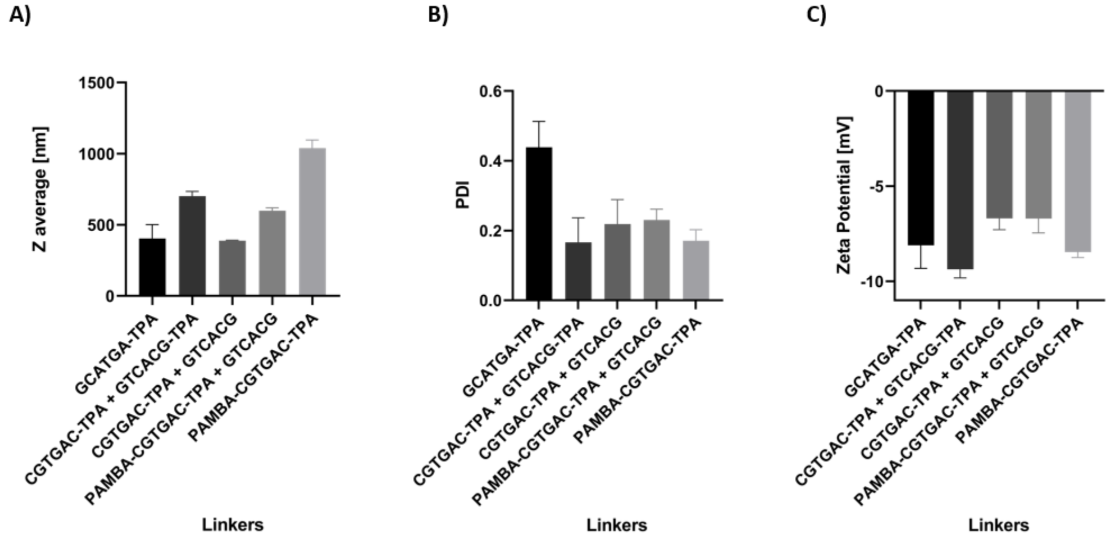

Figure S1. Dynamic light scattering (DLS) and electrophoretic light scattering (ELS) of PNA-Zr particles. Linkers are specified below each bar. A) Z-average in nanometers (measured in water). B) Polydispersity index (measured in water). C) Zeta potential in millivolt (measured in 10 mM NaCl).

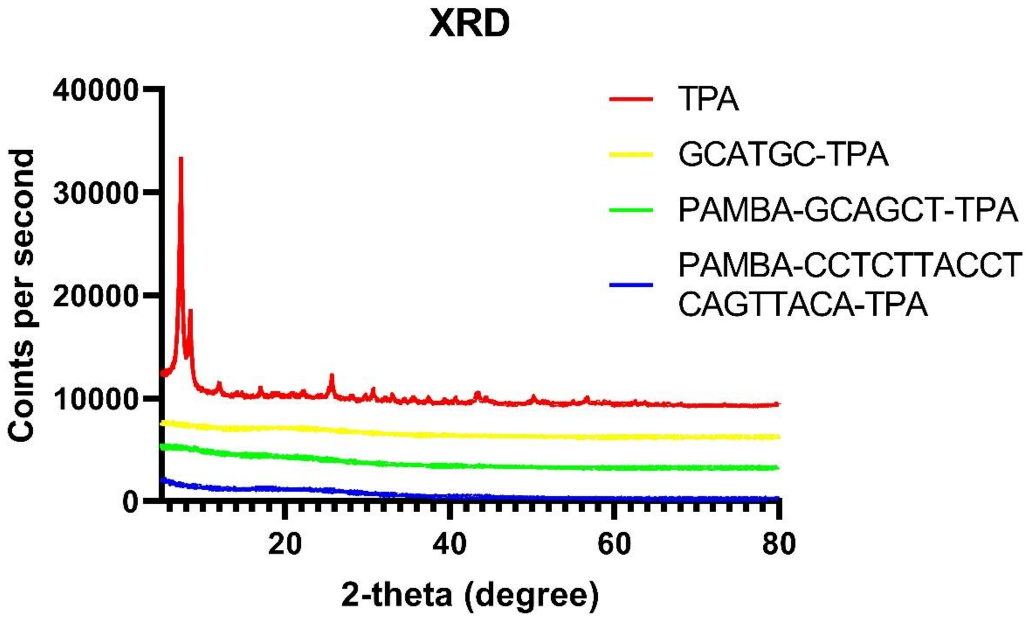

Figure S2. X-ray diffraction of metal-organic nanoparticles based on different linkers: TPA (red), double-stranded palindromic GCATGC-TPA (yellow), single-stranded PAMBA-GCAGCT-TPA (green) and splice-switching PAMBA-CCTCTTACCTCAGTTACA-TPA (blue). TPA curve was shifted by 9000 counts, GCATGC-TPA curve by 6000 counts and PAMBA-GCAGCT-TPA by 3000 counts to enable direct comparison.

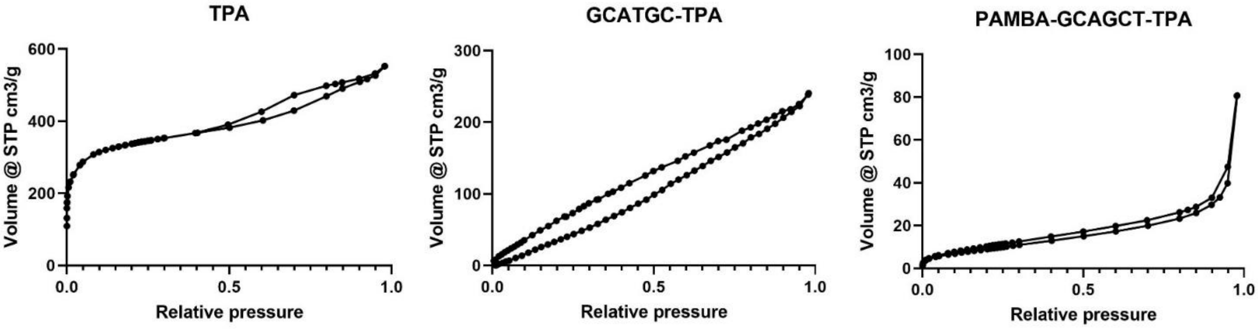

Figure S3. Nitrogen sorption isotherms of TPA- (UiO-66) and PNA-Zr particles.

Table S3. Nitrogen sorption results of PNA-Zr particles. Surface areas of the samples were calculated according to the linearized Brunauer–Emmett–Teller (BET) equation.

Linkers	Correlation coefficient	C constant	Surface Area (m^2/g)
TPA	r =0.999850	356.959	1260.791
GCATGC-TPA	r = 0.999352	2.683	300.533
PAMBA-GCAGCT-TPA	r =0.999454	28.079	35.845

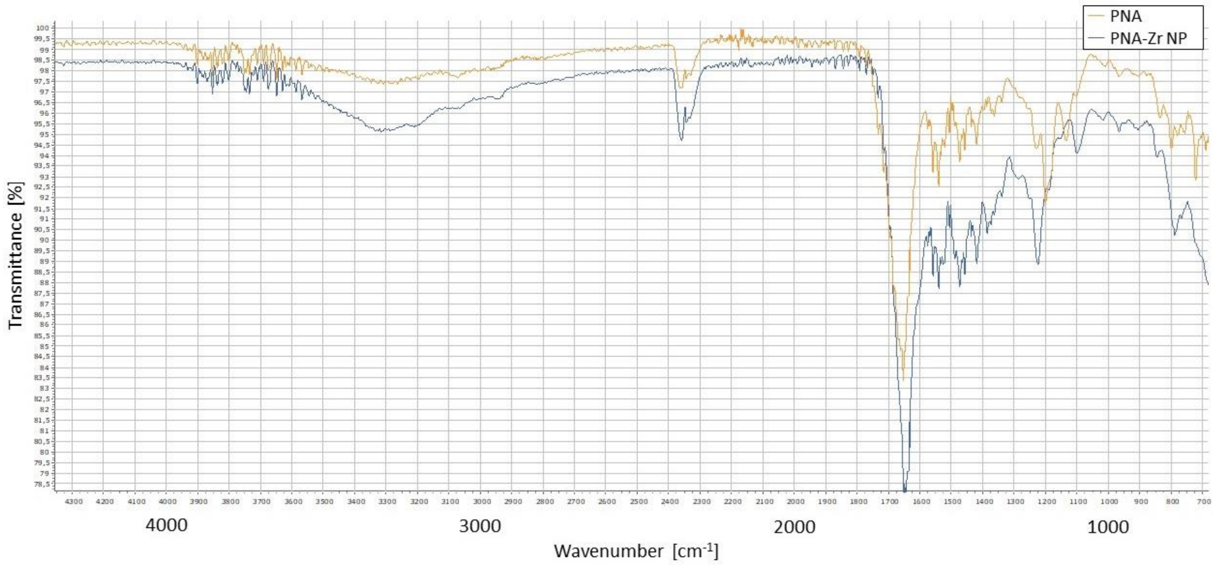

Figure S4. FT-IR spectra of 705 SSO PNA (orange) and 705 SSO PNA-Zr particles (blue).

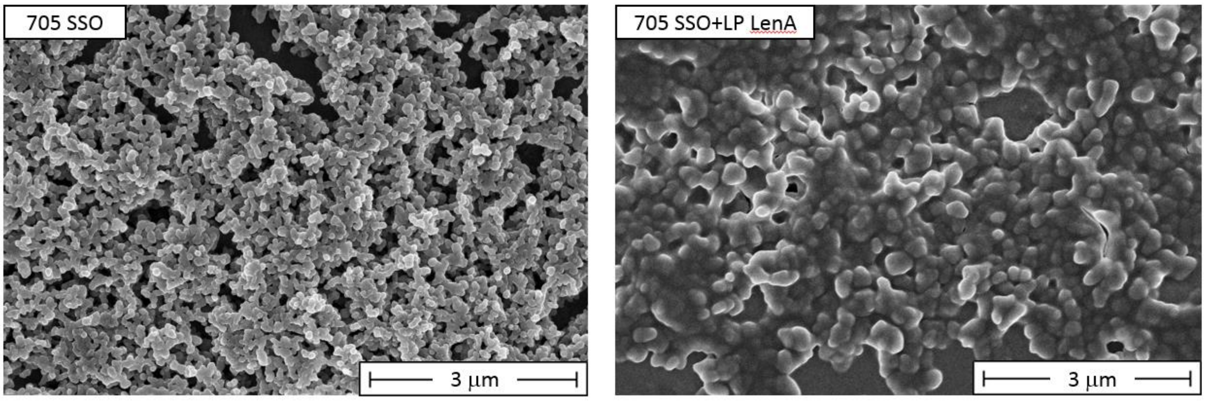

Figure S5. Representative SEM images of PNA-Zr particles with 705 SSO linker and without (left) or with (right) LP LenA coating. (Scale bars = 3 μm).

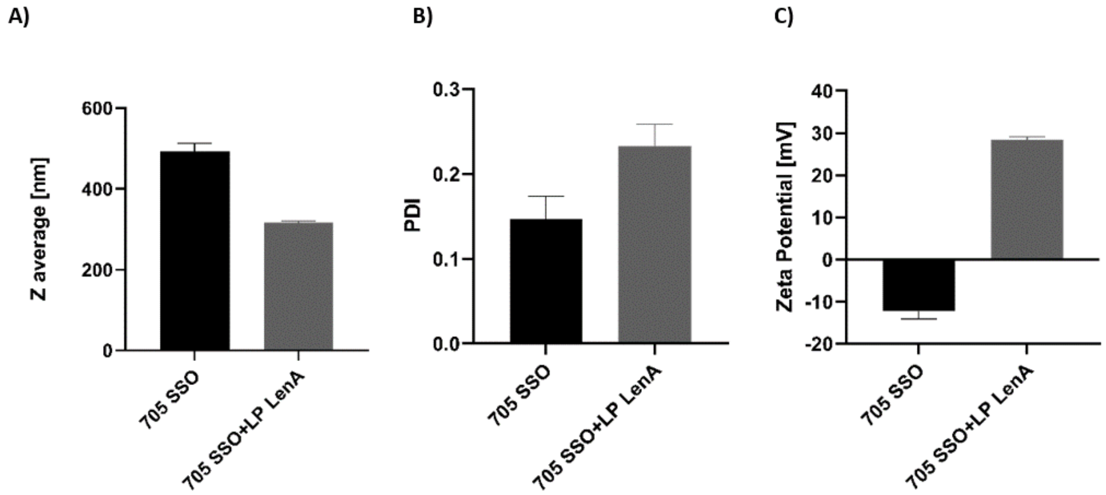

Figure S6. Dynamic light scattering (DLS) and electrophoretic light scattering (ELS) of 705 SSO-Zr particles with or without LP LenA coating. Linkers are specified below each bar. A) Z-average in nanometers (measured in water). B) Polydispersity index (measured in water). C) Zeta potential in millivolt (measured in 10 mM NaCl).

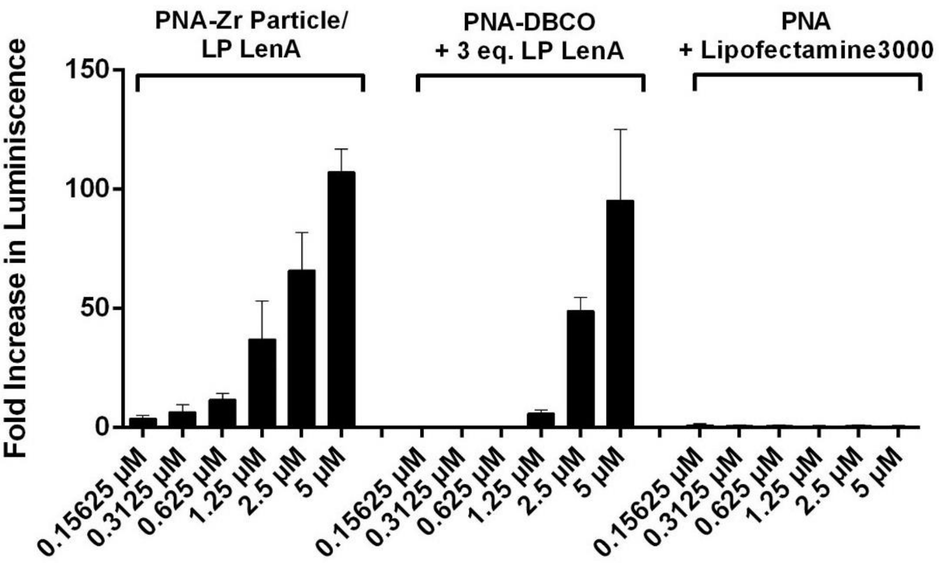

Figure S7. Splice-switching activity of different 705 SSO PNA formulations in HeLA pLuc/705 cells. PNA-Zr particles were coated with LP LenA at a ratio of 1/1.25. DBCO-modified 705 SSO PNA derivative was conjugated with 3 eq. of azide-containing LP LenA. Lipofectamine 3000 was used according to the instructions by the manufacturer for the transfection of 200 ng pDNA per well. All luciferase activity assays were carried out in triplicates.

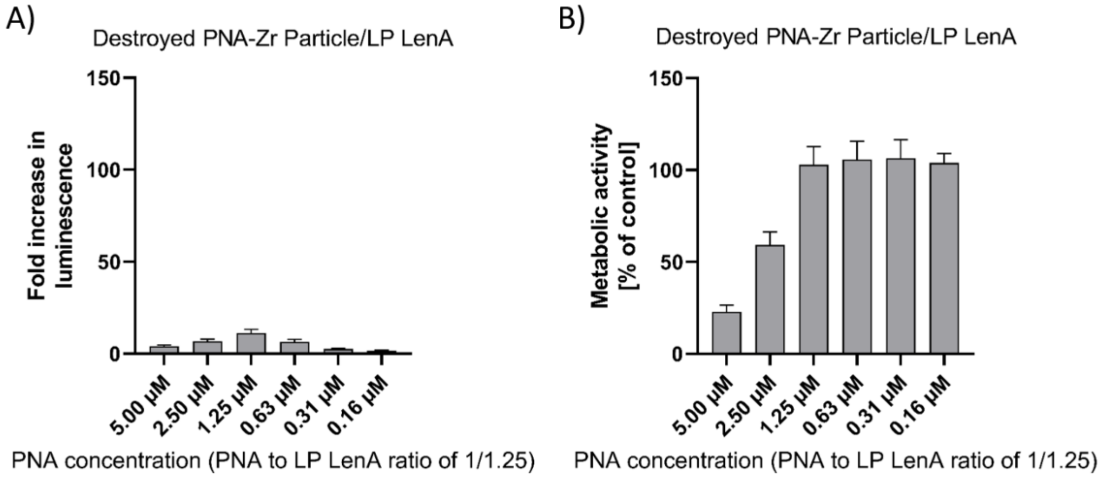

Figure S8. A) Splice-switching activity of destroyed PNA-Zr particles containing SSO 705 in HeLa pLuc/705 cells. Cells were treated for 48 h with solutions of disassembled particles containing 0.16 to 5 µM SSO 705 and a 1:1.25 PNA to LP LenA ratio before luciferase activity assay. B) Metabolic activity was determined by CellTiter-Glo® assay in HeLa wild type cells after 48 hours treatment with destroyed particles containing 0.16 to 5 µM PNA and a 1:1.25 PNA to LP LenA ratio. Metabolic activity was determined as percentage of luminescence levels in treated cells normalized to HBG treated control cells.

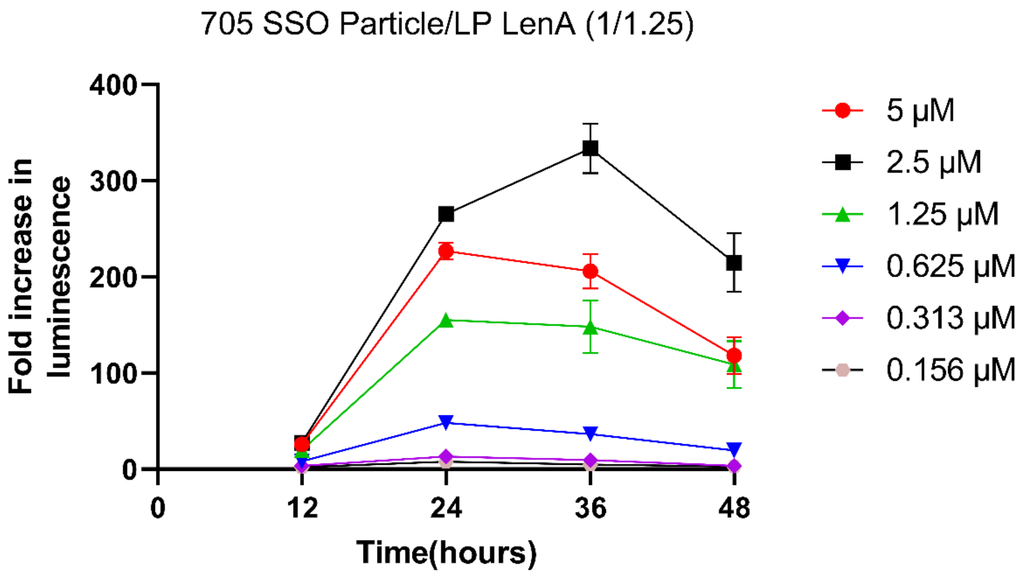

Figure S9. Splice-switching activity of LP LenA coated 705 SSO-Zr nanoparticles in HeLA pLuc/705 cells. PNA concentrations are indicated with different colors (fixed ratio of 1/1.25 PNA/LP LenA). Incubation was terminated after the indicated time points, followed by luciferase activity assay. All luciferase activity assays were carried out in triplicates.

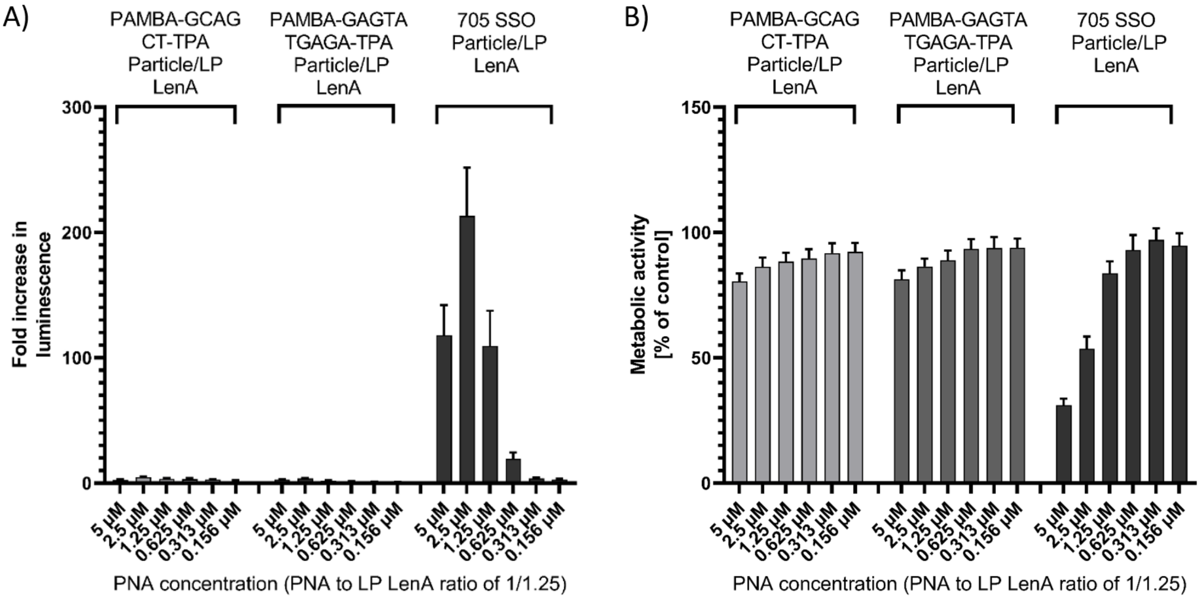

Figure S10. Splice-switching activity and metabolic activity of PNA-Zr nanoparticles containing different PNA sequences (controls or functional 705 SSO). A) Splice-switching activity at different PNA concentrations (fixed ratio of 1/1.25 PNA/LP LenA) in HeLA pLuc/705 cells. B) Metabolic activity of HeLa wild type cells at different PNA concentrations (fixed ratio of 1/1.25 PNA/LP LenA). All luciferase and metabolic activity assays were carried out in triplicates. 705 SSO was used as positive control.

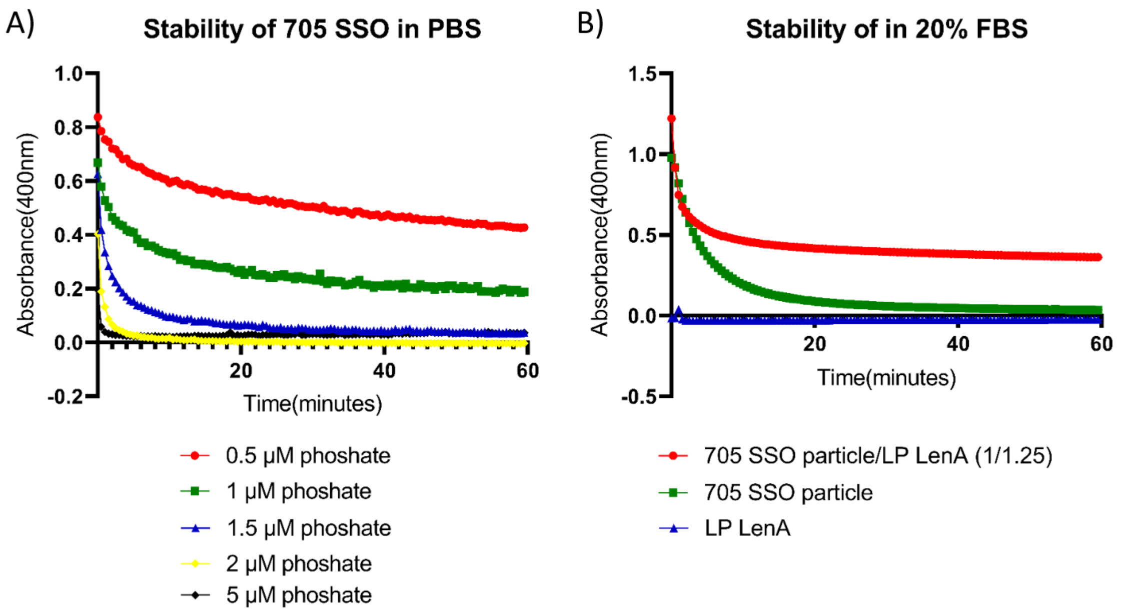

Figure S11. Stability of 705 SSO-Zr particles determined by measurement of optical density (400nm) in PBS and FBS. A) Stability of 705 SSO-Zr particles at PBS dilutions leading to different phosphate concentrations as indicated by colors. B) Stability of 705 SSO-Zr particles (red), LP LenA coated 705 SSO-Zr particles (green) in 20% filtered FBS. Free LP LenA was used as control to exclude unspecific particle formation between the cationic lipopeptide and serum proteins.

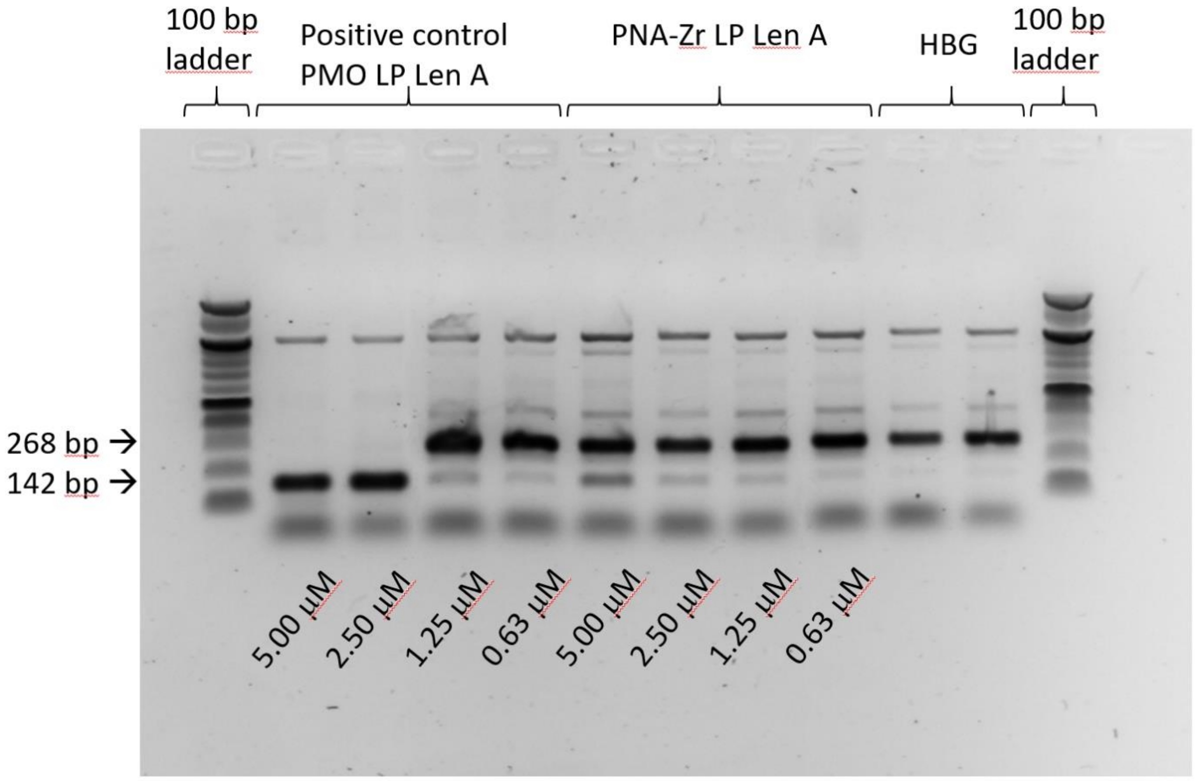

Figure S12. RT-PCR of β-globin IVS2 from HeLa pLuc/705 cells treated with PNA-Zr/LP LenA at ratio 1/1.25. Total RNA was isolated 48 h after treatments and β-globin IVS2 was amplified. Band at 268 bp represents the aberrant splicing product; band at 142 bp corresponds to the product after splice-switch. HBG and 705 SSO PMO conjugated to LP Len A (cf. Kuhn et al.) were used as negative and positive controls, respectively.

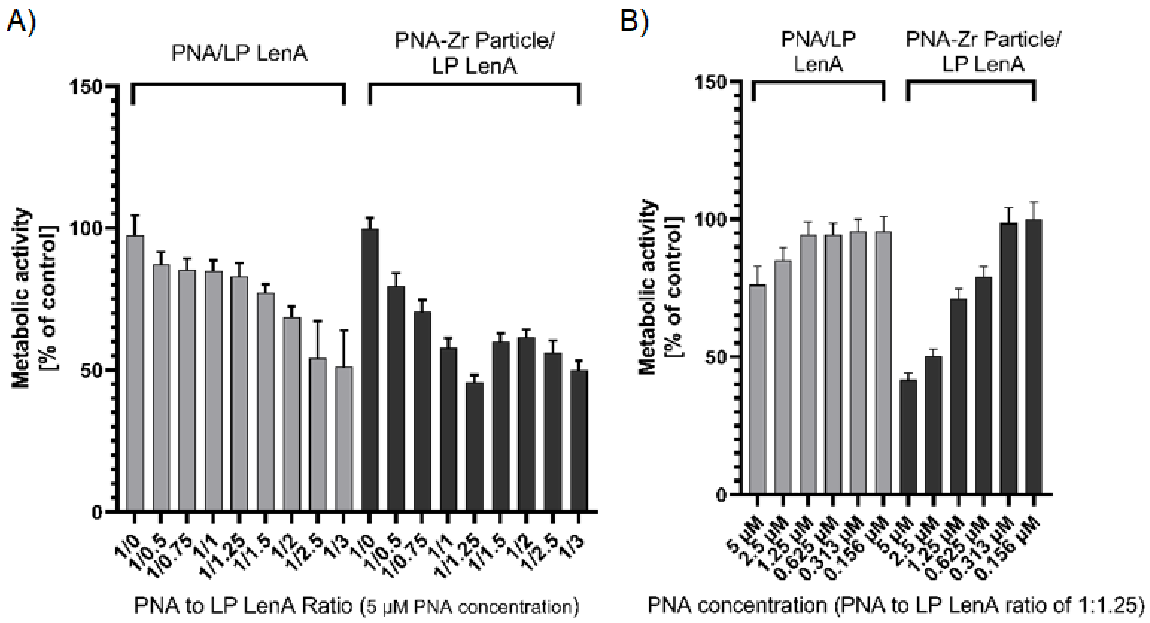

Figure S13. Metabolic activity of HeLa wild type cells as determined by CellTiter-Glo® assay after treatment with **A)** 705 SSO-Zr nanoparticles (5 µM PNA) at different PNA/LP LenA coating ratios. Free 705 SSO with or without LP LenA was used as a control; **B)** 705 SSO-Zr nanoparticles (1/1.25 PNA/LP LenA ratio) at different PNA concentrations. All luciferase and metabolic activity assays were carried out in triplicates.

3. Analytical data

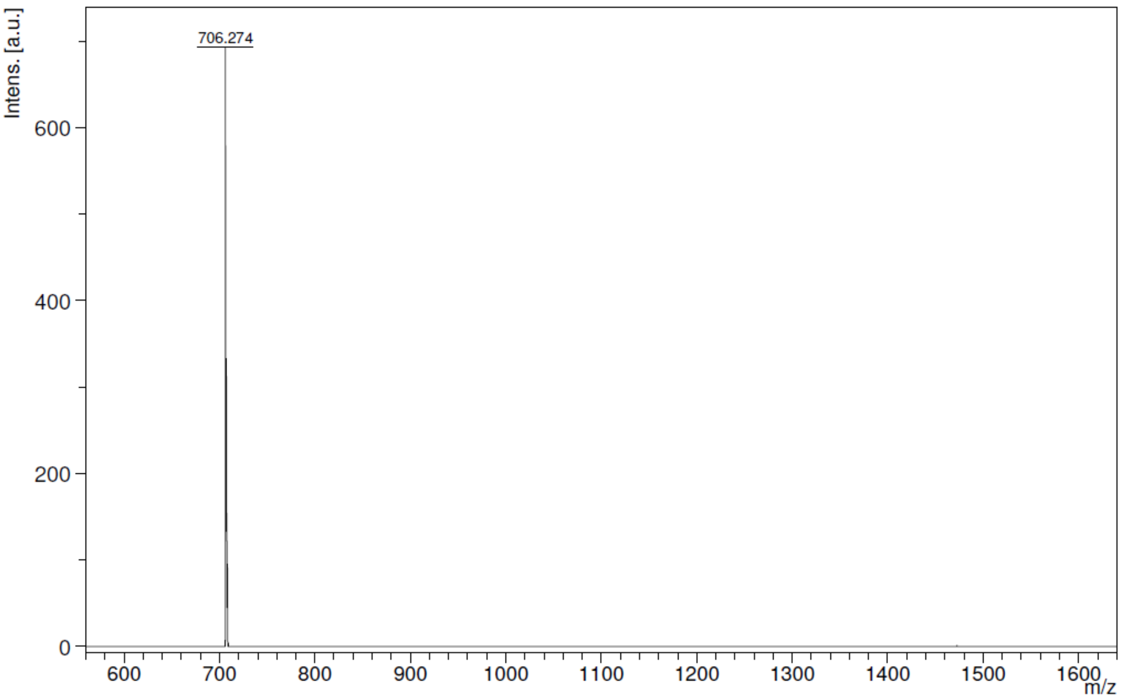

Figure S14. MALDI-TOF-MS spectrum of GC-TPA (calculated 707.5, observed 706.3).

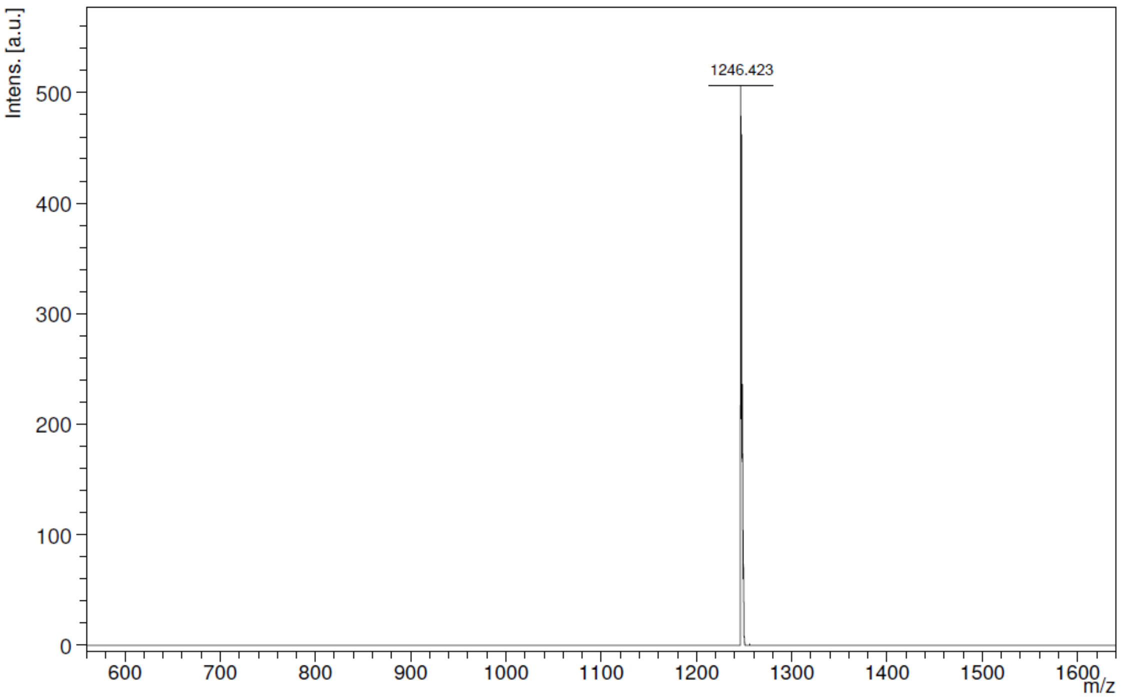

Figure S15. MALDI-TOF-MS spectrum of CATG-TPA (calculated 1249.1, observed 1246.4).

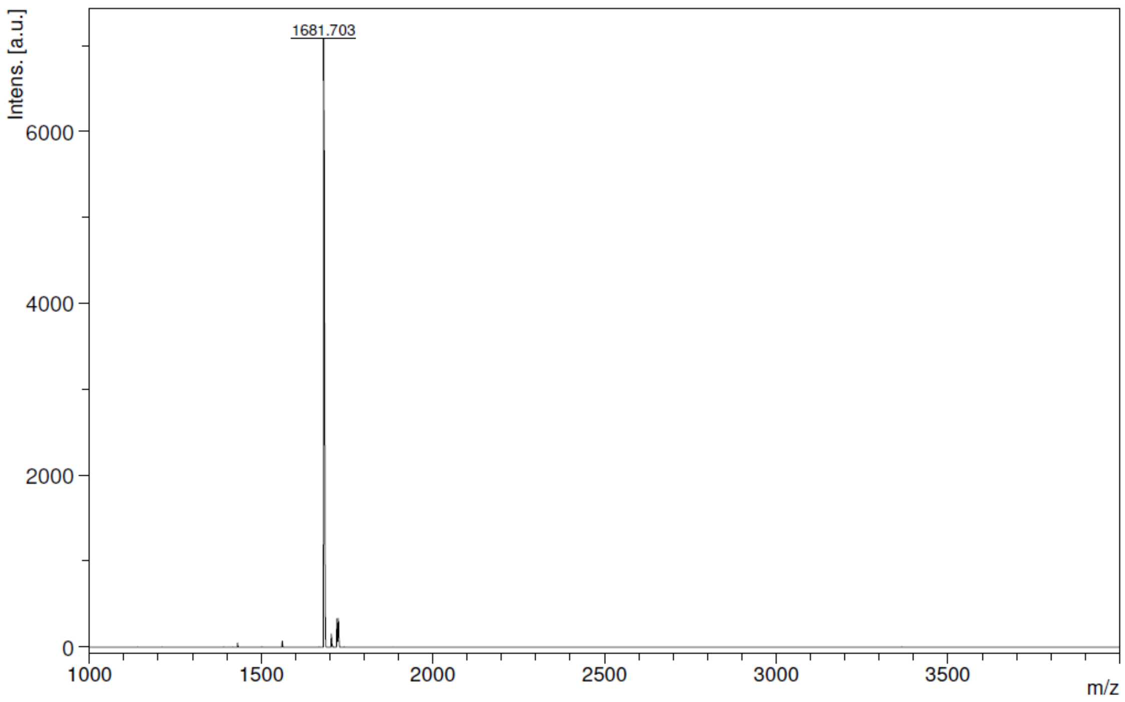

Figure S16. MALDI-TOF-MS spectrum of GCATGC-ACI (calculated 1685.6, observed 1681.7).

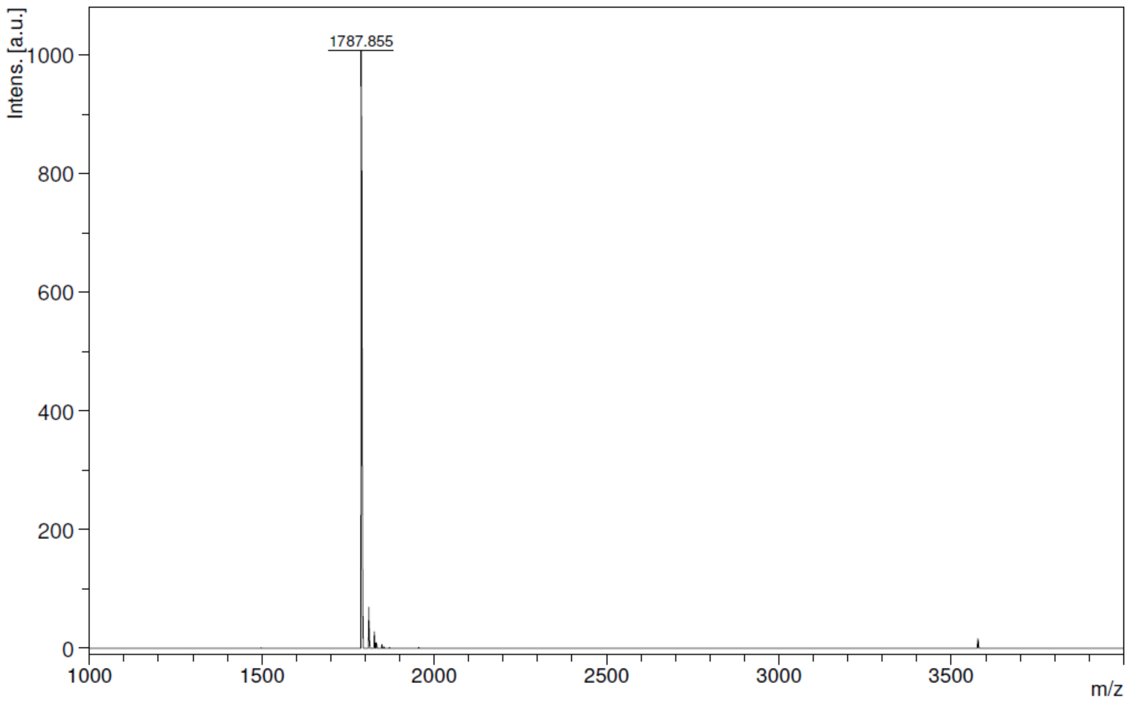

Figure S17. MALDI-TOF-MS spectrum of GCATGC-TPA (calculated 1791.6, observed 1787.9).

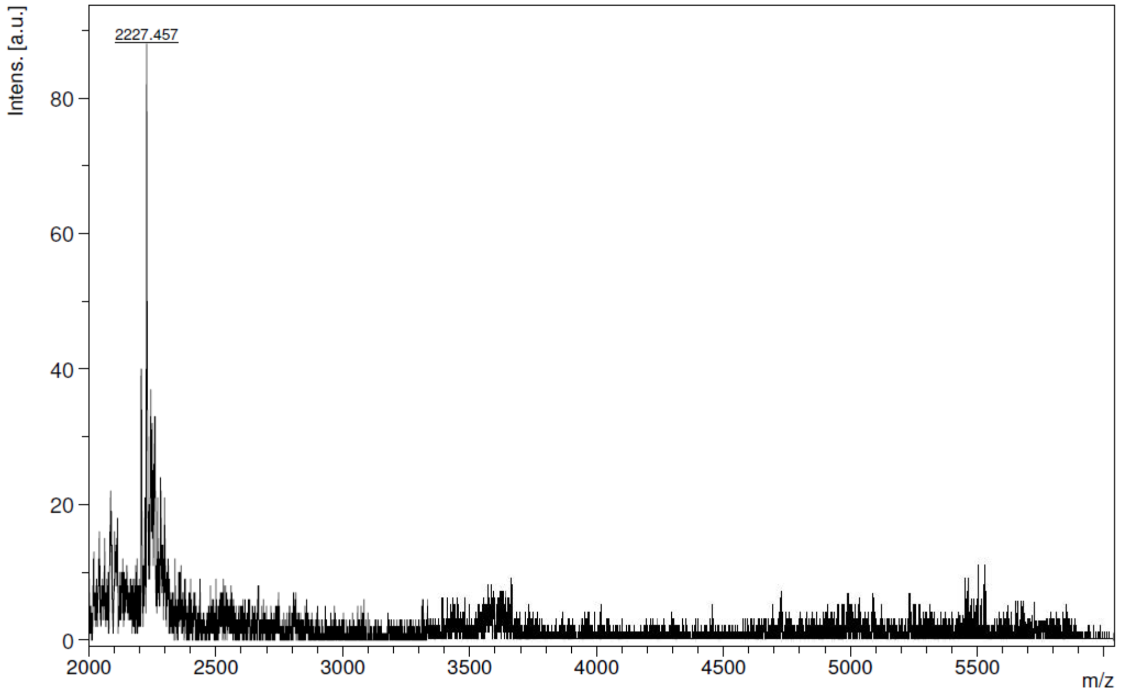

Figure S18. MALDI-TOF-MS spectrum of CAGTACTG-ACI (calculated 2227.1, observed 2227.5).

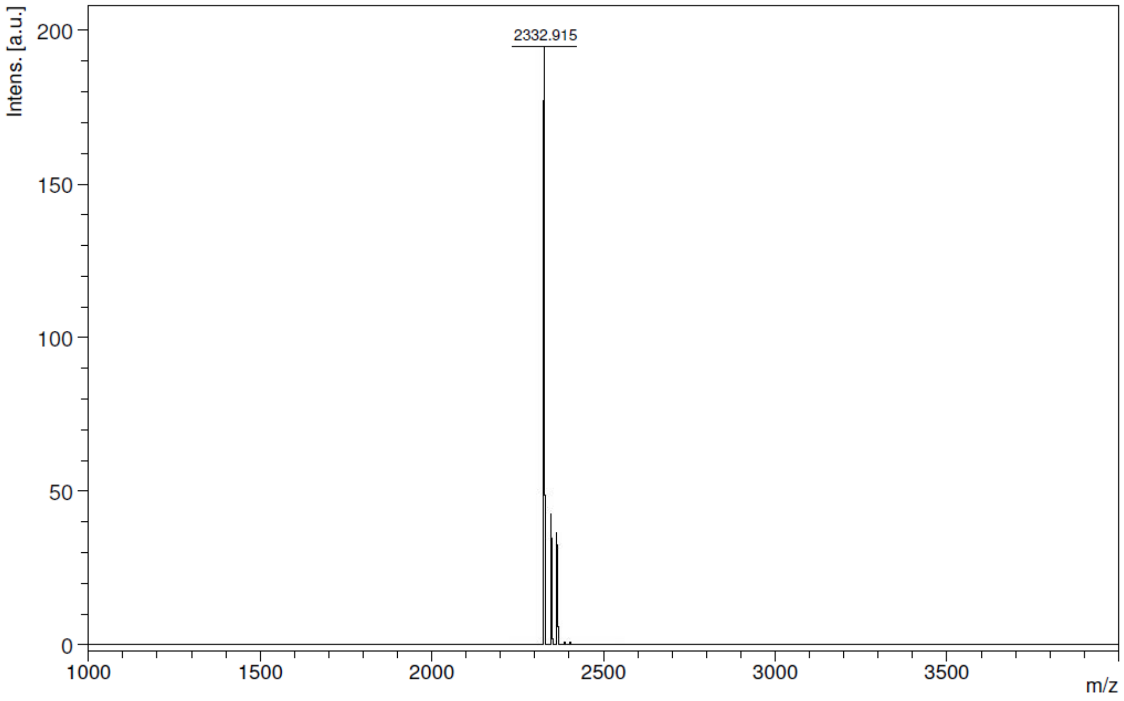

Figure S19. MALDI-TOF-MS spectrum of CAGTACTG-TPA (calculated 2333.1, observed 2332.9).

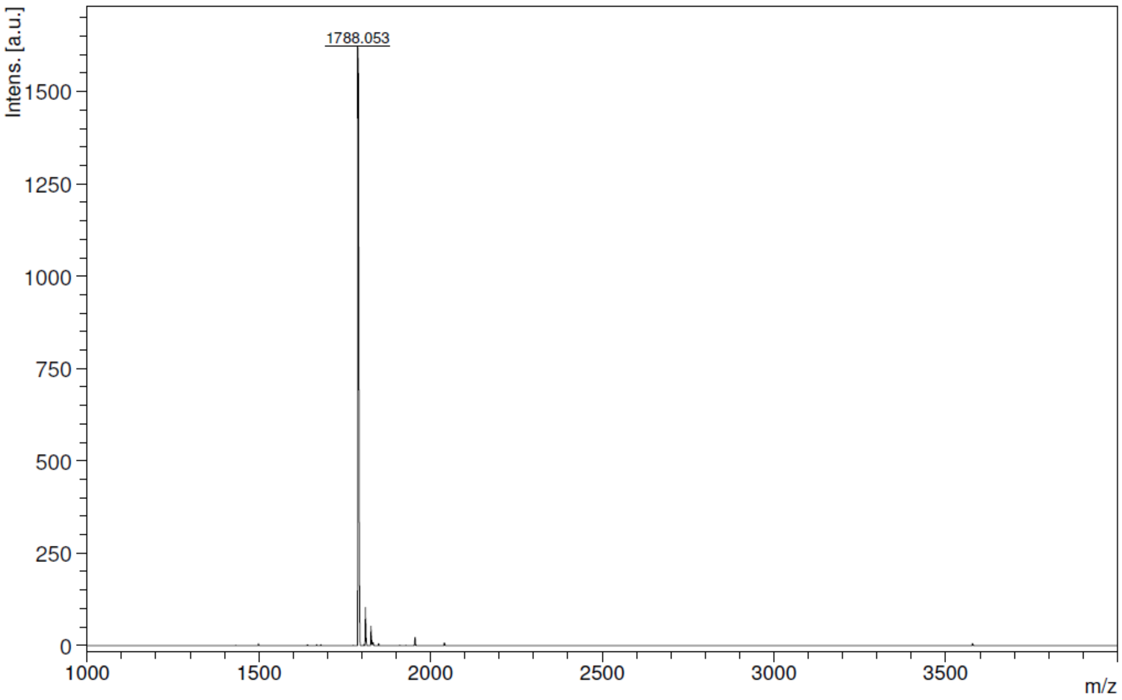

Figure S20. MALDI-TOF-MS spectrum of CGTGAC-TPA (calculated 1791.6, observed 1788.1).

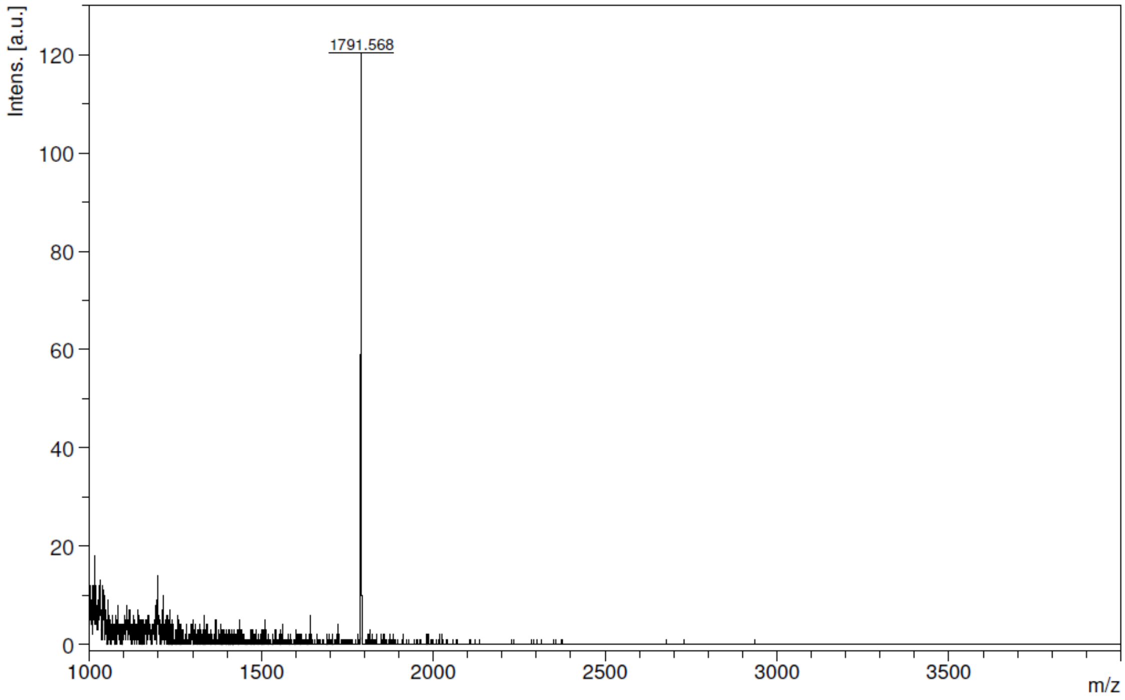

Figure S21. MALDI-TOF-MS spectrum of GTCACG-TPA (calculated 1791.6, observed 1791.6).

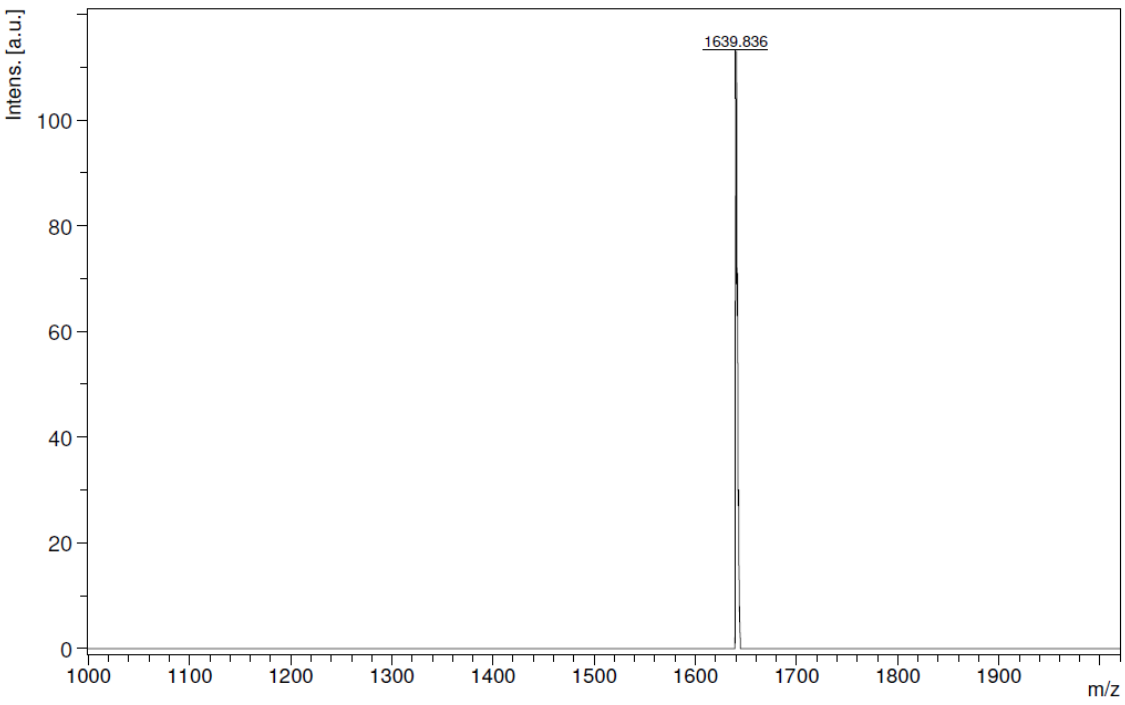

Figure S22. MALDI-TOF-MS spectrum of GTCACG (calculated 1643.6, observed 1639.8).

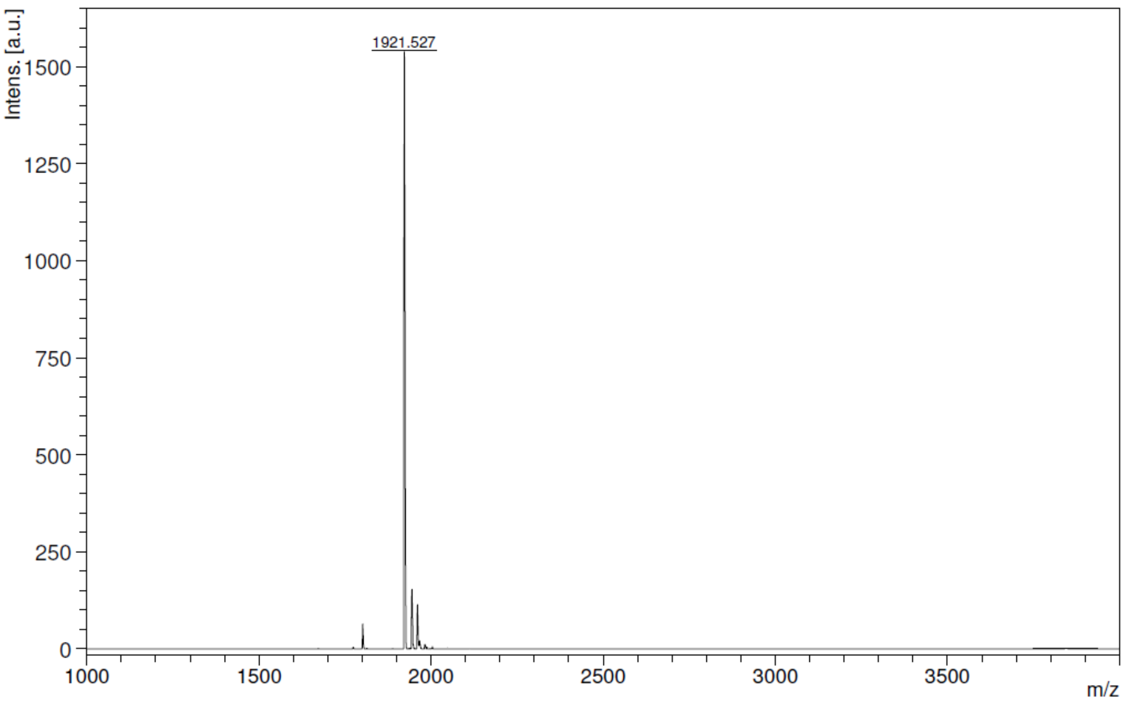

Figure S23. MALDI-TOF-MS spectrum of PAMBA-CGTGAC-TPA (calculated 1924.6, observed 1921.5).

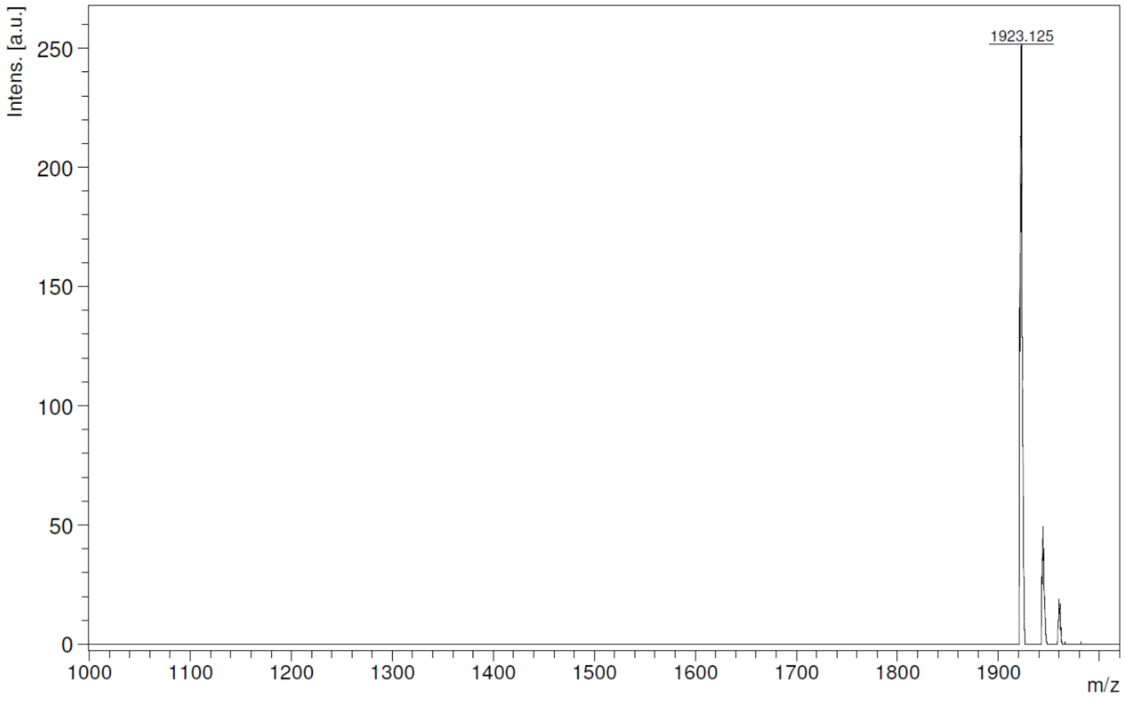

Figure S24. MALDI-TOF-MS spectrum of PAMBA-GCAGCT-TPA (calculated 1924.6, observed 1923.1).

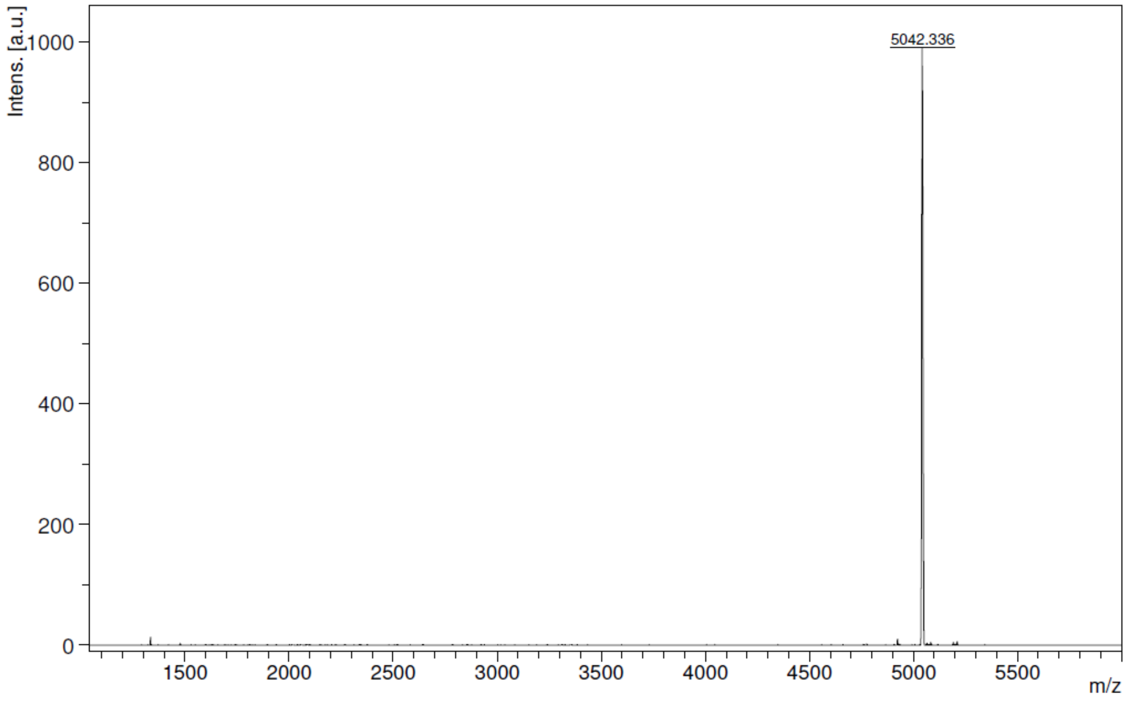

Figure S25. MALDI-TOF-MS spectrum of PAMBA-CCTCTTACCTCAGTTACA-TPA (calculated 5046.6, observed 5042.3).

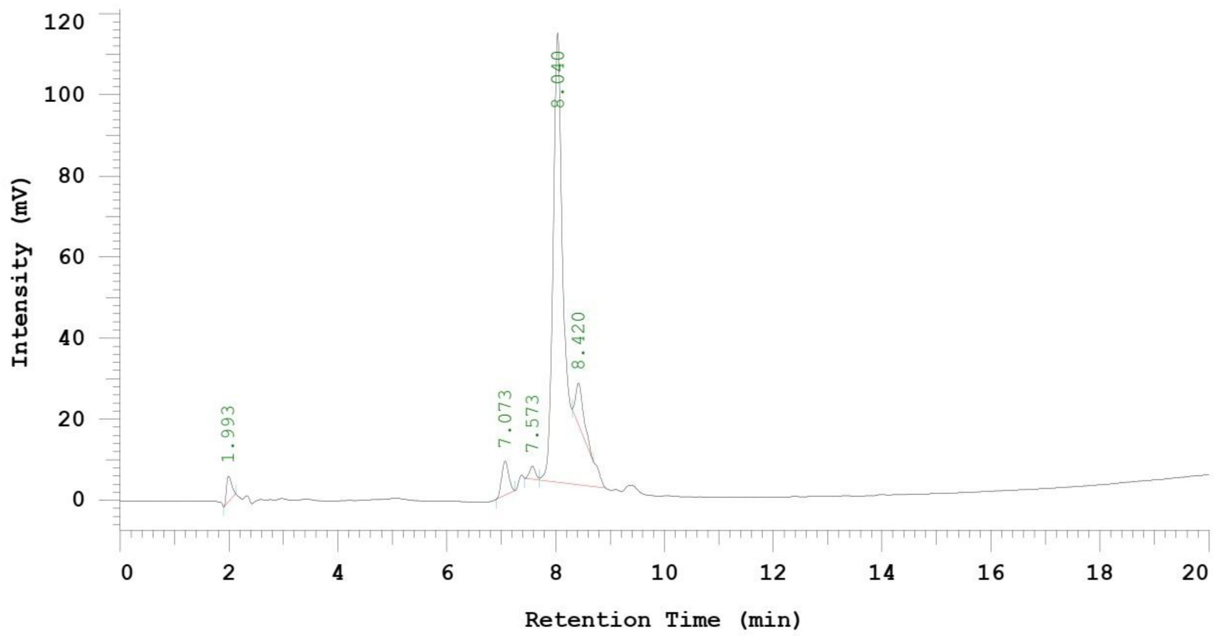

Figure S26. RP-HPLC chromatogram of GTCACG-TPA. A VWR-Hitachi Chromaster HPLC system (5160 Pump System, 5260 Autosampler, 5430 Diode Array Detector; VWR, Darmstadt, Germany) equipped with a YMC RP-18 column and a gradient from 5% acetonitrile to 100% acetonitrile (each containing 0.1 % TFA) in 15 min and detection at 254 nm were used.

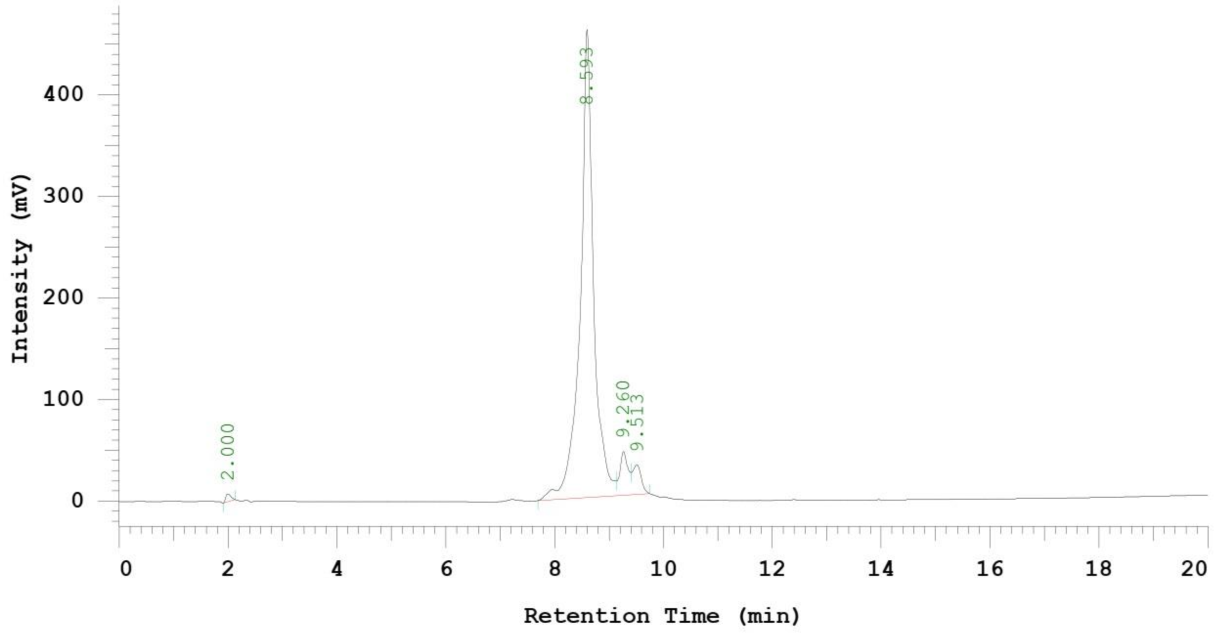

Figure S27. RP-HPLC chromatogram of PAMBA-GCAGCT-TPA. A VWR-Hitachi Chromaster HPLC system (5160 Pump System, 5260 Autosampler, 5430 Diode Array Detector; VWR, Darmstadt, Germany) equipped with a YMC RP-18 column and a gradient from 5% acetonitrile to 100% acetonitrile (each containing 0.1 % TFA) in 15 min and detection at 254 nm were used.

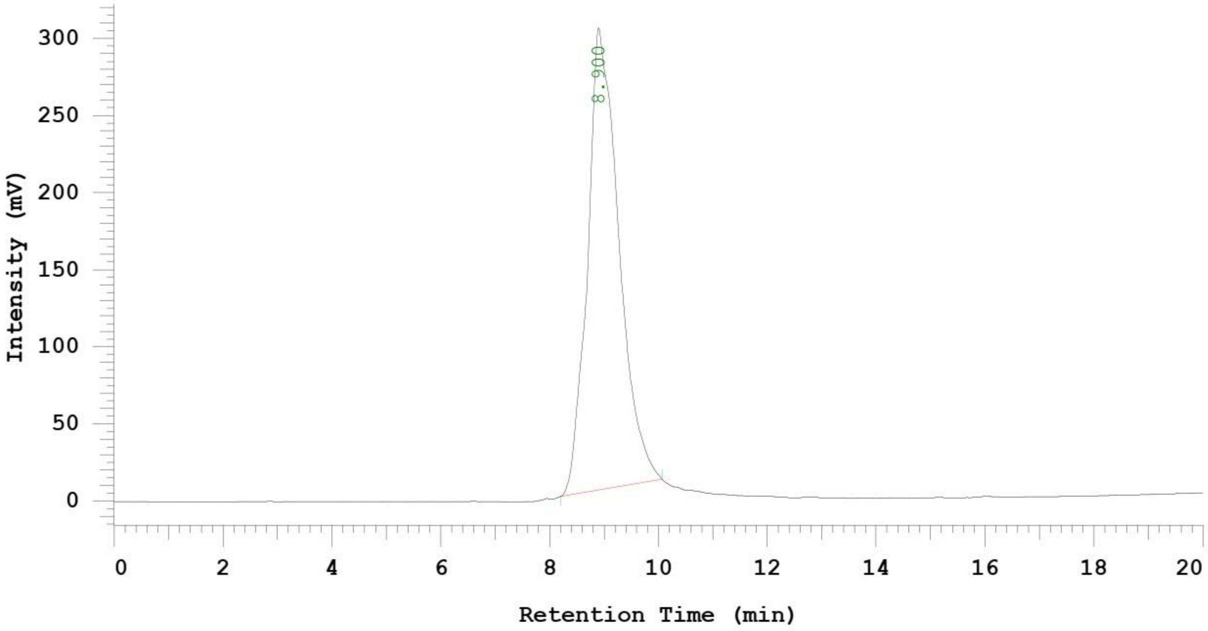

Figure S28. RP-HPLC chromatogram of PAMBA-CCTCTTACCTCAGTTACA-TPA. A VWR-Hitachi Chromaster HPLC system (5160 Pump System, 5260 Autosampler, 5430 Diode Array Detector; VWR, Darmstadt, Germany) equipped with a YMC RP-18 column and a gradient from 5% acetonitrile to 100% acetonitrile (each containing 0.1 % TFA) in 15 min and detection at 254 nm were used.

4. References

J. Kuhn, P. M. Klein, N. Al Danaf, J. Z. Nordin, S. Reinhard, D. M. Loy, M. Höhn, S. El Andaloussi, D. C. Lamb, E. Wagner, Y. Aoki, T. Lehto and U. Lächelt, *Advanced Functional Materials*, 2019, **29**, 1906432.
